# Scalable and Practical Approach of Phenol Formation from Hydroxylation of Arylboronic Acids under Metal-, Photocatalyst-, and Light-Free Conditions

**DOI:** 10.1021/acsomega.4c02844

**Published:** 2025-01-13

**Authors:** Shuqing Cai, Noreen Rehmat, Zafar Mahmood, Qian Chen, Yanping Huo, Shaomin Ji

**Affiliations:** † School of Chemical Engineering and Light Industry, Guangdong University of Technology, Guangzhou 510006, P. R. China; ‡ Guangdong Provincial Laboratory of Chemistry and Fine Chemical Engineering Jieyang Center, Jieyang 515200, P. R. China

## Abstract

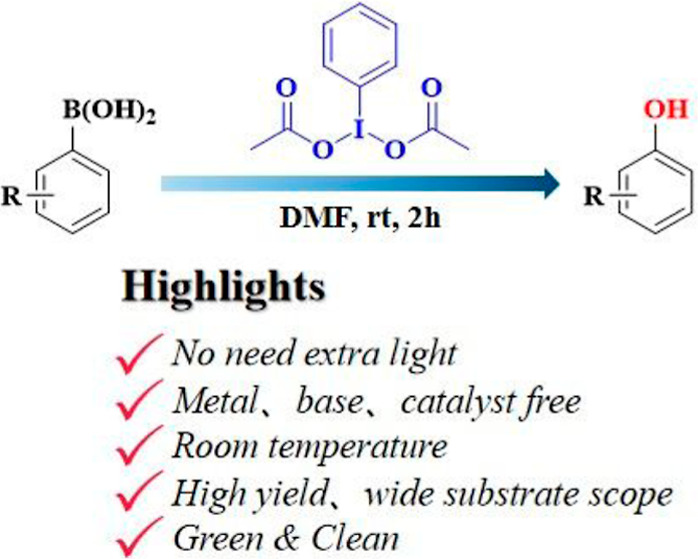

Phenols are of particular importance owing to their widespread application in various fields, including life sciences and synthetic chemistry. However, the typical synthetic strategies of phenol either rely on metal- or photo-catalysts or involve stringent conditions such as high temperature, strong oxidants, and acidic or basic environments. Herein, we present a remarkably simple, ecofriendly, and photocatalyst-free synthesis of phenols. In contrast to conventional methods, which relies on expensive catalysts or hazardous reagents, the current diacetoxyiodobenzene-mediated hydroxylation of arylboronic acid protocol offers significant improvement in terms of costs, operational simplicity, toxicity, and sustainability. The reaction does not require high temperature or light activation and shows good functional group compatibility and conversion efficiency (55–96%). Moreover, these mild reaction conditions are also useful for gram-scale synthesis without compromising the reaction yield.

## 1 Introduction

Phenols and its derivatives are a ubiquitous constituent of various biological active compounds, natural products, pharmaceuticals, and agrochemicals.^1−4^ They also serve as important building blocks in the various industries including perfume, textile, and material, as well as act as a versatile synthetic intermediate in the construction of functional polymers and heterocycles.^5,6^ Due to their excellent antioxidant, anti-inflammatory, antiseptic, and antibacterial properties, phenols are widely used in the treatment of various diseases as well as in several biological activities.^7^ For instance, the aerosol form of phenol is used for the treatment of sore throat, while tolcapone, penicillin, amoxicillin, and vanillic acid, all containing the phenol scaffold, are commonly used in the treatment of the Parkinson disease,^8^ as anesthesia, and as a flavoring dye, respectively.^9^ In brief, phenols are an amazing structural motif that spread extensively in the whole natural world, even being part of the human body.^10^ Consequently, due to its high demand in several areas, establishing a facile development method that ensures the efficient access to phenols is of great significance.

In the past, most of the phenol was derived from coal tar and petroleum, while its substitute derivatives were prepared adopting the various multistep transformation approaches.^11−13^ The conventionally most practiced strategies to form the phenol derivatives are (i) Pd-catalyzed transformation and nucleophilic aromatic substitution of activated aryl halides,^14^ leading to the conversion of the C–X bond into C–O bond;^15−19^ (ii) metal-catalyzed hydrolysis and transformation of diazoarenes,^20^ transformation of the C–M bond into C–O bond;^15,21^ and (iii) the direct oxidation of the C–H bond into C–O bond.^22−24^ However, the harsh reaction conditions, high cost of the expensive metal catalyst, narrow substrate scope, and poor compatibility with several function groups make these approaches less attractive and unfavorable for large-scale synthesis.

**Scheme 1:**
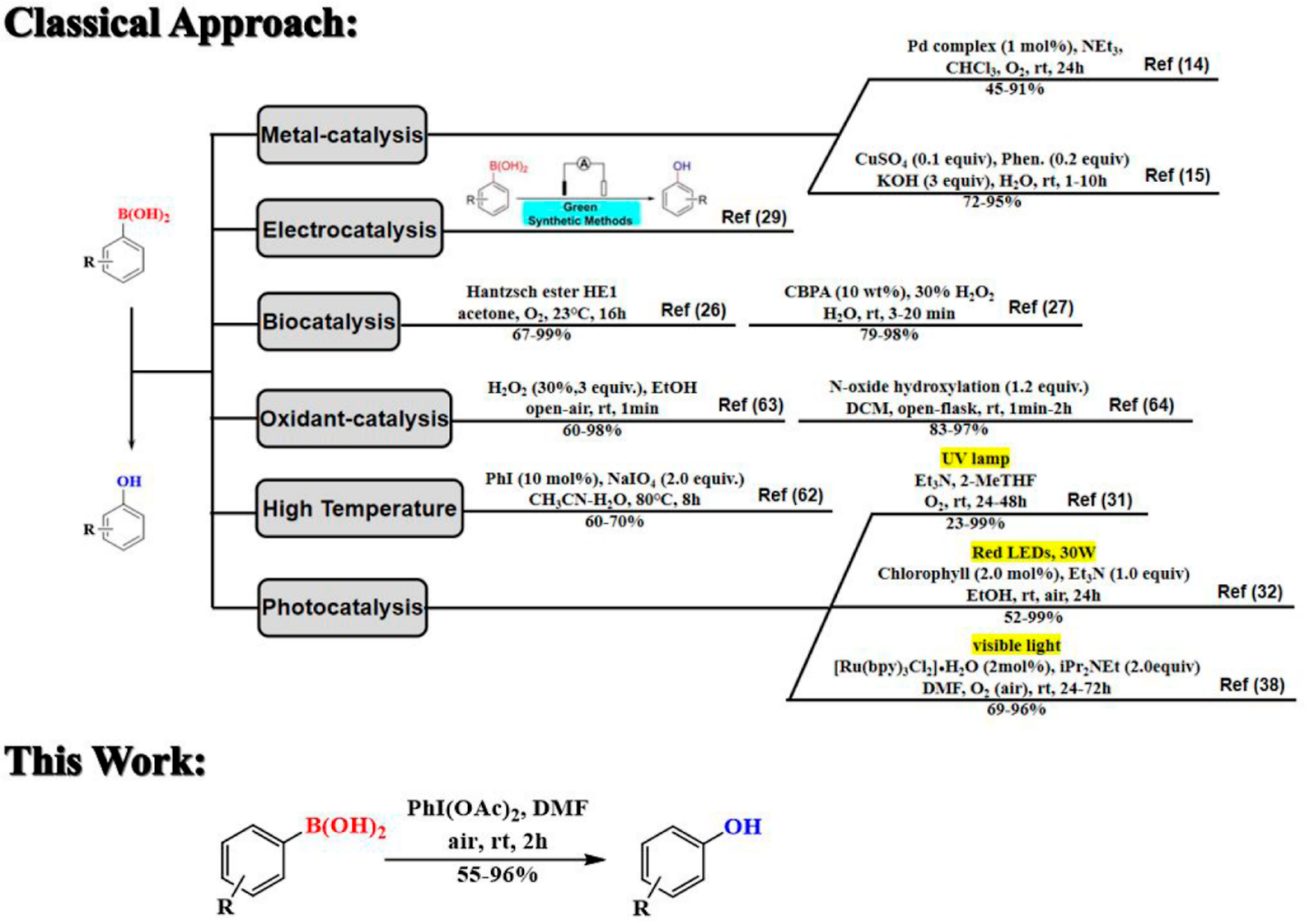
Comparison of the Current Work with Conventional Approaches for the Synthesis of Phenols from Arylboronic Acids

In recent years, the synthesis of phenol from arylboronic acids (Scheme 1) has gained special attention due to several reasons.^25−31^ For instance, arylboronic acids are versatile building blocks that are readily available, and more than four thousand precursors are known. In addition, the less toxic nature, good stability toward heat, moisture, and air, and facile conversion into phenols compared to that of arylamines and halides have made them a valuable precursor; and several methods for their oxidation have been developed.^32−36^ For instance, Xiao and Jørgensen^37^ demonstrated the first example of arylboronic acid oxidation into phenols using the transition metal catalyst *Ru(bpy)*_3_^*2+*^. This was a great achievement, but involvement of a precious metal complex makes this approach unfavorable for commercial synthesis. Considering this, Pitre et al. reported the similar visible light photoredox oxidation protocol of arylboronic acid using methylene blue as the photocatalyst.^38^ Later, numerous photocatalysts, including quantum dots,^39^ metal organic frameworks,^40−42^ cationic polycarbazole networks, and purely organic photocatalysts such as Eosin Y along with the PhI(OAc)_2_ oxidant, have been employed, and efficient conversion of arylboronic acids to phenols was achieved.^43^ Despite this achievement, the above-mentioned protocols have some limitations such as the high cost due to the use of precious metal catalysts, strong acidic/basic conditions, requirement of a long reaction time, tedious synthesis of photocatalysts, or limited substrate scope.^26,27,31,44−48^ Further, such protocols involving harsh reaction conditions and metal impurities are also not preferred for pharmaceutical purpose.^14,49−54^

Nowadays, several alternative metal-free aerobic oxidation approaches for the hydroxylation of arylboronic acid are being developed,^28,30,31,55,56^ for instance, the hydroxylation of arylboronic acids using oxidants such as hydrogen peroxide,^26,56^ oxone,^57^*N*-oxides,^58^ molecular oxygen,^59^ hypervalent iodine reagent, and so on.^60^ These approaches make the oxidation of arylboronic acids under simple and metal-free conditions possible, but in many of these methods, an excess amount of oxidants is used, which may cause the overoxidation of several sensitive functional groups; thus, very careful conditions are required.^61−66^ Moreover, some protocols work under basic conditions or at high temperature, which is also harmful.^44,60^ Further, reports on nonmetal-mediated oxidation of arylboronic acids with scaled-up demonstration are rare, and this research area is still under active investigation.^66^ Therefore, the development of a new mild and affective approach for the conversion of arylboronic acids to phenols is desired.

To address the above problems, herein, we report a simple, efficient, and straightforward practical approach to prepare phenols from the aryl boronic acids. This transformation is accomplished with high yield under mild conditions using diacetoxyiodobenzene [DIB; PhI(OAc)_2_] as a mediator, and the method features the following: (i) Requiring no transition metal catalyst or photocatalyst; (ii) requiring no light irradiation, operating under dark and mild conditions with high conversion efficiency; (iii) requiring no harsh conditions and operating at room temperature; and (iv) scalability of the reaction up to gram and large scale. Noticeably, the reaction involves one equivalent of the inexpensive PhI(OAc)_2_ mediator and has good tolerance of different functional groups, showing the suitability and efficiency of the proposed method.

## 2 Results and Discussion

DIB (diacetoxyiodobenzene), a hypervalent iodine reagent, is widely used in organic synthesis as an oxidant due to its accessibility and nontoxic nature,^67,68^ but mainly under thermal conditions. Inspired by these studies and to further expand the scope of this chemistry, we decided to explore the potential of PhI(OAc)_2_ in the transformation of aryl(boronic) acids into corresponding phenols.

We commenced our work to obtain the optimal reaction conditions taking phenyl boronic acid as a model substrate. The screening of other oxidants including DDQ, DMP, PIFA, and PhI(OAc)_2_ (Table 1, entries 1–4) suggested that PhI(OAc)_2_ was the most effective. The initial survey of various solvents (Table 1, 4–7 entries) showed that the transformation of phenyl boronic acid proceeded in both the protic and nonprotic solvents, but the conversion efficiency was found to be sensitive to the solvent nature.

**Table 1:**
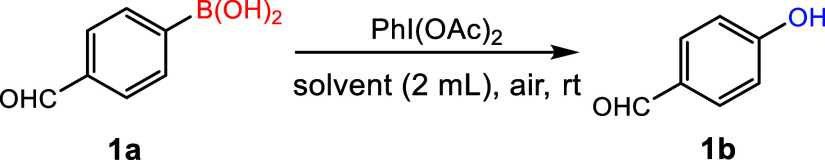
Optimization of the Hydroxylation of Arylboronic Acids^a^

entry	oxidant	solvent	yield(%)
1	1 equiv. DDQ	DMF	83
2	1 equiv. DMP	DMF	63
3	1 equiv. PIFA	DMF	75
4	1 equiv. PhI(OAc)_2_	DMF	96
5	1 equiv. PhI(OAc)_2_	ACN	35
6	1 equiv. PhI(OAc)_2_	THF	68
7	1 equiv. PhI(OAc)_2_	DMSO	84
8	0.5 equiv. PhI(OAc)_2_	DMF	52
9	0.25 equiv. PhI(OAc)_2_	DMF	20
10	0	DMF	0
11^b^	1 equiv. PhI(OAc)_2_	DMF	50
12^b^	4 equiv. PhI(OAc)_2_	DMF	90

a Reaction conditions: **1a** (0.2 mmol) and anhydrous solvent (2 mL) were stirred in an air atmosphere under the dark conditions for 2 h at room temperature.

b Under a N_2_ atmosphere.

From the preliminary experiments, we found that dimethylformamide (DMF) is the best solvent, in which a high yield of up to 96% was observed at room temperature in just 2 h. Note that the solubility of PhI(OAc)_2_ is found to be comparatively better in DMF and dimethyl sulfoxide (DMSO) than that in acetonitrile (ACN) under the given conditions, which is one probable reason for high yield in these solvents compared to that in ACN. In addition, to optimize the minimum concentration of the PhI(OAc)_2_ oxidant, a series of reactions were conducted (Table 2, 4, 8–10 entries), and the product separation was performed using the standard column chromatography technique. It was noticed that just 1 equiv of PhI(OAc)_2_ is sufficient to ensure the maximum conversion of phenyl boronic acid into phenol. Use of further high PhI(OAc)_2_ loading does not improve the yield, while a decrease in its concentration significantly lowers the yield.

**Table 2:**
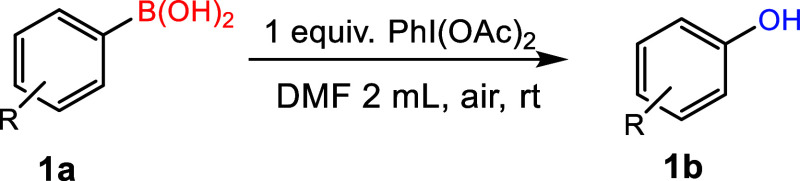

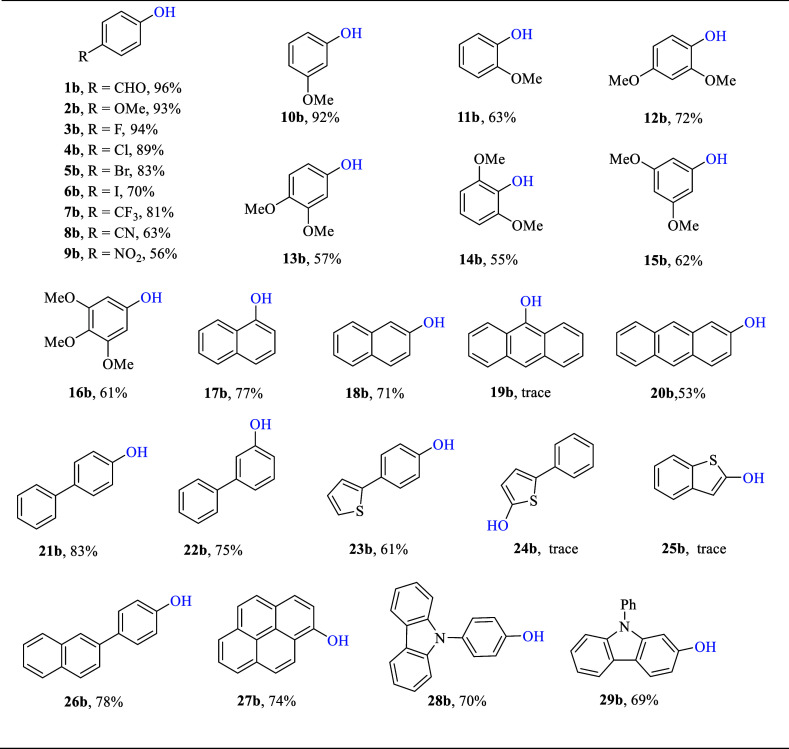
Substrate Expansion of Aryboronic Acids^a^

a Reaction conditions: **1a** (0.2 mmol) and diacetoxyiodobenzene (0.2 mmol), stirred at room temperature for 2 h in 2 mL of DMF solvent under dark conditions; isolated yield based on **1a**.

Keeping the standard reaction conditions in hand, next, the versatility and scope of the proposed research methodology for the transformation of various phenyl boronic acids bearing different functional groups were examined (Table 2). A variety of phenyl boronic acids with various substituents were smoothly converted under the optimized conditions into corresponding phenols in good to excellent yield (55–96%), proving the compatibility of the proposed methodology with a wide range of substrates. We found that the electron-withdrawing or electron-donating substituents, such as OMe, F, Cl, CN, etc., have a negligible effect on the reaction efficiency as both electron-poor and electron-rich arylboronic acids showed efficient conversion into the desired product (yield: 63–96%). However, heterocyclic boronic acids were found to be incompatible with this approach as negligible product was obtained under the present conditions. Notably, substitution of various halogen atoms (F, Cl, Br, and I) can be tolerated, which can further be functionalized to afford the complex functional organic compounds. In addition, the current protocol was also found to be suitable for the conversion of polysubstituted arylboronic acids, and boronic acid appended to diverse aromatic skeletons such as anthracene, naphthalene, carbazole, pyrene, etc., showed conversion into corresponding phenols in moderate to good yield, proving the good tolerance of the substrate.

Note that the current protocol is useful for selectively functionalizing the -*ortho*, -*meta*, or -*para* position of arenes; all substituted aryls furnished the product in good yield (55–92%). Previous studies revealed that the synthesis of *meta*-substituted arene is challenging compared to functionalization of *ortho*/*para* positions of aryls due to the strong *ortho-*/*para*-activation effect. To our delight, the current strategy is found to be suitable for the synthesis of *meta*-arylated phenols with a high yield (92%). Further, to investigate the practicality, the substrate concentration was extended up to the gram scale (2 mmol), and the corresponding product was obtained under the standard conditions without affecting the reaction yield (Supporting Information).

It is worthy to mention that the current strategy is also benefited by simple and straight workup, and a pure product is obtained by the standard column chromatography technique (see the Supporting Information for the details). Though a number of heterogeneous photocatalytic phenol production systems involving more simple and easier purification are available, these systems inherently require stringent conditions such as photocatalysts and strong light intensity and also involve several steps ,which may results in lower yield. However, this study provides a simple and green phenol synthetic approach.

**Scheme 2:**
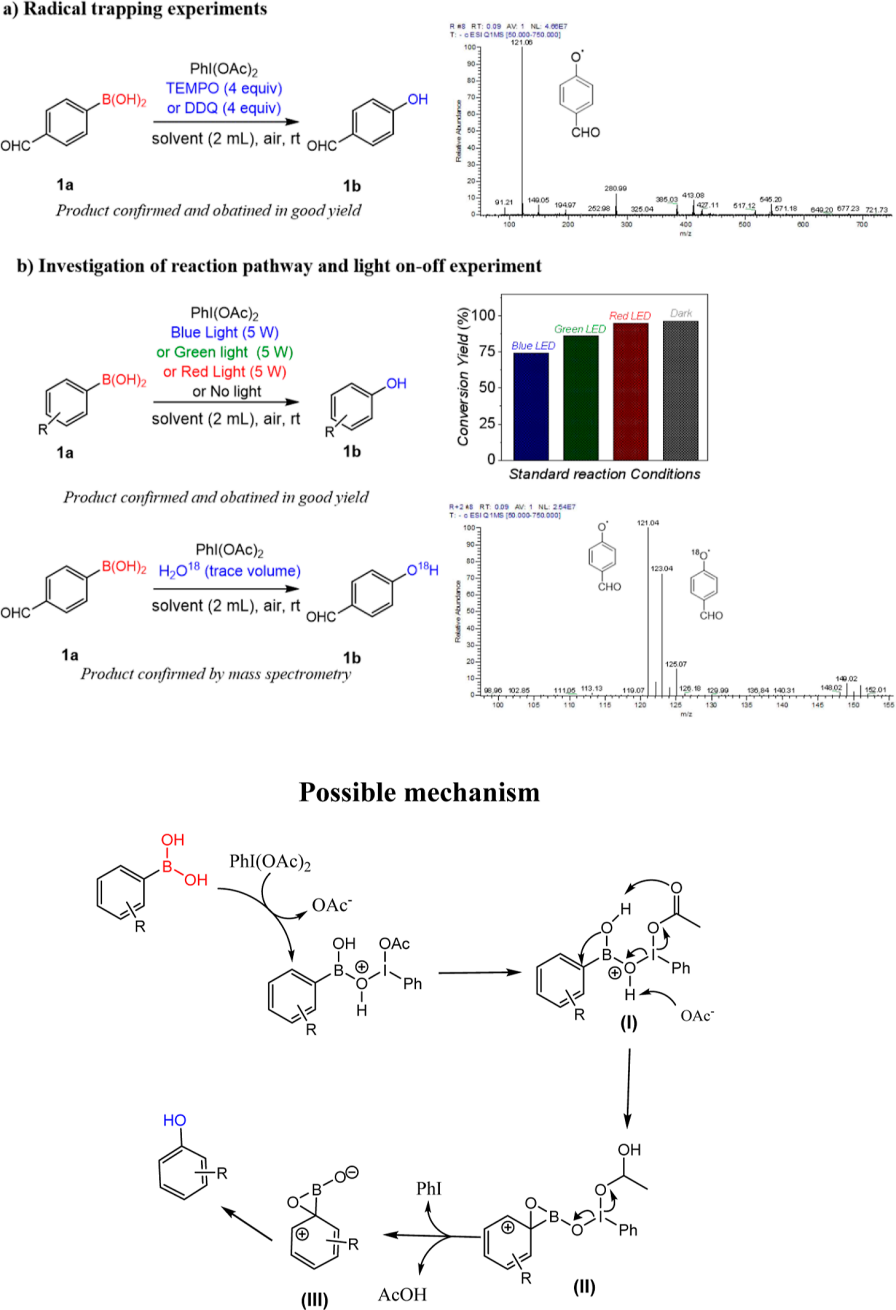
Proposed Plausible Reaction Mechanism of Hydroxylation of Arylboronic Acids

For mechanistic insights into this PhI(OAc)_2_-mediated transformation, several controlled experiments were conducted, which confirmed that conversion can be accomplished without light, air, and photocatalyst (Scheme 2a). Using a typical LED of blue, green, or red color, the light source selection experiment was conducted, showing that the product is formed both in dark and light conditions, which confirmed that it is not a light-driven transformation (Scheme 2b). Our investigation started with phenyl boronic acid as a model substrate, and excellent conversion into corresponding phenol was observed in the presence of PhI(OAc)_2_ upon irradiating the mixture with a blue LED (5 W). For further insights, we perform the reaction using LEDs of various colors (blue, green, red, and dark), and in each case, the product has been obtained (Scheme 2b). Surprisingly, excellent conversion was observed even when the reaction was performed in the dark. We found that the direct transformation of aryl(boronic) acid into corresponding phenols is possible without light and additional photocatalyst, with just stirring in the presence of PhI(OAc)_2_ at room temperature in the dark. The transformation furnished smoothly both in air and under deaerated conditions, which excludes the involvement of oxygen. Further, when the reaction was performed in the presence of 4 equiv. of TEMPO, known as free radical scavengers, the desired product was still obtained in good yield, excluding the involvement of free radicals in this transformation (Scheme 2a). This assumption was further confirmed by an electron paramagnetic resonance study in which no paramagnetic or unpaired intermediate species were detected. For further verification of the involved mechanism, the reaction was repeated by adding heavy oxygen atom (^18^O)-containing water under the optimal reaction conditions, and the phenolic product with ^18^O was obtained (Scheme 2b). The ratio of the ordinary product to heavy oxygen product was approximately 1.2:1. It is inferred that after adding heavy-oxygen water, some hydroxyl groups in the heavy-oxygen water exchange with hydroxyl groups on boric acid and then react according to the above pathway; most arylboronic acids do not exchange but react directly according to the original path.

Based on the literature^43,65^ and our observation, we propose a plausible mechanism of this transformation (Scheme 2). Initially, the arylboronic acid attacks the iodine center of the diacetoxyiodobenzene (DIB) form an intermediate adduct (I), releasing acetic acid. Next, it undergoes deprotonation to form hypervalent iodine species (II), followed by sequential rearrangement to form the tetracoordinated boron intermediate (III). The subsequent hydrolysis or intramolecular aryl shifting leads to the formation of the desired phenolic product along with acetic acid as a byproduct, as previously reported in the literature.^42^

## 3 Conclusions

In summary, we have established a mild, highly efficient, and photocatalyst- and light-free methodology for the synthesis of phenols via hydroxylation of readily available arylboronic acids using the inexpensive diacetoxyiodobenzene [PhI(OAc)_2_] reagent as an initiator. Compared with conventional methods, the current protocol is an environment-friendly, less toxic, cost-effective, and green approach of phenol synthesis with a simple workup, which makes it potentially easier to scale up. The remarkable feature of this protocol is that the reaction operates at room temperature under mild conditions, has good selectivity and functional group tolerance, and can be scaled up to the gram level. To the best of our knowledge, this study presents a unique, green, and inexpensive method for the transformation of arylboronic acids into phenols. Further investigations may be helpful in improving the workup procedure and extending the scope of this protocols for other valuable transformations, which are currently ongoing.

## Supporting Information Available

General experimental methods, ^1^HNMR and ^13^C NMR data, and additional spectra of the compounds (PDF)

## References

[ref1] Vinogradova N.; Vinogradova E.; Chaplygin V.; Mandzhieva S.; Kumar P.; Rajput V. D.; Minkina T.; Seth C. S.; Burachevskaya M.; Lysenko D.; Singh R. K. Phenolic Compounds of the Medicinal Plants in an Anthropogenically Transformed Environment. Molecules 2023, 28, 6322 10.3390/molecules28176322. 37687151 PMC10488847

[ref2] Yu X. N.; Chen S. S.; Wang W. C.; Deng T. S.; Wang H. L. Empowering alkali lignin with high performance in pickering emulsion by selective phenolation for the protection and controlled-release of agrochemical. J. Clean. Prod. 2022, 339, 130769 10.1016/j.jclepro.2022.130769.

[ref3] Scott K. A.; Cox P. B.; Njardarson J. T. Phenols in Pharmaceuticals: Analysis of a Recurring Motif. J. Med. Chem. 2022, 65, 7044–7072. 10.1021/acs.jmedchem.2c00223. 35533692

[ref4] Carson M. C.; Kozlowski M. C. Recent advances in oxidative phenol coupling for the total synthesis of natural products. Nat. Prod. Rep. 2024, 41, 208–227. 10.1039/D3NP00009E. 37294301 PMC10709532

[ref5] Moon J. D.; Sujanani R.; Geng Z. S.; Freeman B. D.; Segalman R. A.; Hawker C. J. Versatile Synthetic Platform for Polymer Membrane Libraries Using Functional Networks. Macromolecules 2021, 54, 866–873. 10.1021/acs.macromol.0c02414.

[ref6] Bashir M. A.; Wei J.; Wang H. F.; Zhong F. R.; Zhai H. B. Recent advances in catalytic oxidative reactions of phenols and naphthalenols. Org. Chem. Front. 2022, 9, 5395–5413. 10.1039/D2QO00758D.

[ref7] Abdelshafy A. M.; Belwal T.; Liang Z.; Wang L.; Li D.; Luo Z. S.; Li L. A comprehensive review on phenolic compounds from edible mushrooms: Occurrence, biological activity, application and future prospective. Crit. Rev. Food Sci. Nutr. 2022, 62, 6204–6224. 10.1080/10408398.2021.1898335. 33729055

[ref8] Bernatoniene J.; Jakstas V.; Kopustinskiene D. M. Phenolic Compounds of Rhodiola rosea L. as the Potential Alternative Therapy in the Treatment of Chronic Diseases. Int. J. Mol. Sci. 2023, 24, 12293 10.3390/ijms241512293. 37569669 PMC10418374

[ref9] El-Saied G. M. S.; El-Safty O. M. T.; Mohamed A. K. A. M.; Wahab W. A. M. M. A. Comparative Evaluation of the Efficacy of Treatment with Intravenous Lignocaine and Intravenous Granisetron in Prevention of Pain Due to Intravenous Injection of Propofol. QJM-Int J Med 2023, 116, hcad069.024 10.1093/qjmed/hcad069.024.

[ref10] Mena P.; Crozier A. Do (Poly)phenols Matter for Nutrition Research? News from the Front. Mol. Nutr. Food Res. 2022, 66, 2200617 10.1002/mnfr.202200617. 36331114

[ref11] Xiong W. Z.; Shi Q.; Liu W. H. Simple and Practical Conversion of Benzoic Acids to Phenols at Room Temperature. J. Am. Chem. Soc. 2022, 144, 15894–15902. 10.1021/jacs.2c07529. 35997485

[ref12] Wang S. K.; Chen M. T.; Zhao D. Y.; You X.; Luo Q. L. Iodine-Catalyzed Oxidative Aromatization: A Metal-Free Concise Approach to meta-Substituted Phenols from Cyclohex-2-enones. Adv. Synth. Catal. 2016, 358, 4093–4099. 10.1002/adsc.201600930.

[ref13] Gai H. J.; Qiao L.; Zhong C. Y.; Zhang X. W.; Xiao M.; Song H. B. A solvent based separation method for phenolic compounds from low-temperature coal tar. J. Clean. Prod. 2019, 223, 1–11. 10.1016/j.jclepro.2019.03.102.

[ref14] Chowdhury A. D.; Mobin S. M.; Mukherjee S.; Bhaduri S.; Lahiri G. K. [Pd(L)Cl2]-Catalyzed Selective Hydroxylation of Arylboronic Acids to Phenols. Eur. J. Inorg. Chem. 2011, 2011, 3232–3239. 10.1002/ejic.201100240.

[ref15] Xu J. M.; Wang X. Y.; Shao C. W.; Su D. Y.; Cheng G. L.; Hu Y. F. Highly Efficient Synthesis of Phenols by Copper-Catalyzed Oxidative Hydroxylation of Arylboronic Acids at Room Temperature in Water. Org. Lett. 2010, 12, 1964–1967. 10.1021/ol1003884. 20377271

[ref16] Xia S.; Gan L.; Wang K.; Li Z.; Ma D. Copper-Catalyzed Hydroxylation of (Hetero)aryl Halides under Mild Conditions. J. Am. Chem. Soc. 2016, 138, 13493–13496. 10.1021/jacs.6b08114. 27682010

[ref17] Schulz T.; Torborg C.; Schäffner B.; Huang J.; Zapf A.; Kadyrov R.; Börner A.; Beller M. Practical Imidazole-Based Phosphine Ligands for Selective Palladium-Catalyzed Hydroxylation of Aryl Halides. Angew. Chem., Int. Ed. 2009, 48, 918–921. 10.1002/anie.200804898. 19105168

[ref18] Anderson K. W.; Ikawa T.; Tundel R. E.; Buchwald S. L. The selective reaction of aryl halides with KOH: Synthesis of phenols, aromatic ethers, and benzofurans. J. Am. Chem. Soc. 2006, 128, 10694–10695. 10.1021/ja0639719. 16910660

[ref19] Liu W. B.; Yang X. B.; Gao Y.; Li C. J. Simple and Efficient Generation of Aryl Radicals from Aryl Triflates: Synthesis of Aryl Boronates and Aryl Iodides at Room Temperature. J. Am. Chem. Soc. 2017, 139, 8621–8627. 10.1021/jacs.7b03538. 28578579

[ref20] Sergeev A. G.; Schulz T.; Torborg C.; Spannenberg A.; Neumann H.; Beller M. Palladium-Catalyzed Hydroxylation of Aryl Halides under Ambient Conditions. Angew. Chem., Int. Ed. 2009, 48, 9391 10.1002/anie.200990250. 19739153

[ref21] Sunderhaus J. D.; Lam H.; Dudley G. B. Oxidation of carbon-silicon bonds: The dramatic advantage of strained siletanes. Org. Lett. 2003, 5, 4571–4573. 10.1021/ol035695y. 14627386

[ref22] Cheng L.; Wang H. H.; Cai H. R.; Zhang J.; Gong X.; Han W. Iron-Catalyzed Arene C-H Hydroxylation. Synlett 2022, 33, A10–A13. 10.1126/science.abj0731 34591631

[ref23] Börgel J.; Tanwar L.; Berger F.; Ritter T. Late-Stage Aromatic C–H Oxygenation. J. Am. Chem. Soc. 2018, 140, 16026–16031. 10.1021/jacs.8b09208. 30421917

[ref24] Yuan C. X.; Liang Y.; Hernandez T.; Berriochoa A.; Houk K. N.; Siegel D. Metal-free oxidation of aromatic carbon-hydrogen bonds through a reverse-rebound mechanism. Nature 2013, 499, 192–196. 10.1038/nature12284. 23846658

[ref25] Fan C. H.; Xu T. Y.; Ke Z. H.; Yeung Y. Y. Autocatalytic aerobic ipso-hydroxylation of arylboronic acid with Hantzsch ester and Hantzsch pyridine. Org. Chem. Front. 2022, 9, 4091–4096. 10.1039/D2QO00618A.

[ref26] Das S. K.; Tahu M.; Gohain M.; Deka D.; Bora U. Bio-based sustainable heterogeneous catalyst for ipso-hydroxylation of arylboronic acid. Sustainable Chem. Pharm. 2020, 17, 100296 10.1016/j.scp.2020.100296.

[ref27] Obah Kosso A. R.; Sellet N.; Baralle A.; Cormier M.; Goddard J. P. Cyanine-based near infra-red organic photoredox catalysis. Chem. Sci. 2021, 12, 6964–6968. 10.1039/d1sc00998b. 34123323 PMC8153078

[ref28] Fu Z. J.; Yi X. Z.; Fang Z. Q.; Zhong T. T.; He D. D.; Guo S. M.; Cai H. An Electrochemical Method for Deborylative Hydroxylation of Arylboronic Acids under Metal-free Conditions. Chem.—Asian J. 2022, 17, 6 10.1002/asia.202200780. 36279188

[ref29] Gualandi A.; Savoini A.; Saporetti R.; Franchi P.; Lucarini M.; Cozzi P. G. A facile hydroxylation of arylboronic acids mediated by sodium ascorbate. Org. Chem. Front. 2018, 5, 1573–1578. 10.1039/C8QO00061A.

[ref30] Xu Y. T.; Li C. Y.; Huang X. B.; Gao W. X.; Zhou Y. B.; Liu M. C.; Wu H. Y. Photoinduced hydroxylation of arylboronic acids with molecular oxygen under photocatalyst-free conditions. Green Chem. 2019, 21, 4971–4975. 10.1039/C9GC02229E.

[ref31] Yan P.; Zeng R.; Bao B.; Yang X. M.; Zhu L.; Pan B.; Niu S. L.; Qi X. W.; Li Y. L.; Ouyang Q. Red light-induced highly efficient aerobic oxidation of organoboron compounds using spinach as a photocatalyst. Green Chem. 2022, 24, 9263–9268. 10.1039/d2gc03055a.

[ref32] Yang S. A.; Li P. H.; Wang Z. H.; Wang L. Photoinduced Oxidative Formylation of N,N-Dimethylanilines with Molecular Oxygen without External Photocatalyst. Org. Lett. 2017, 19, 3386–3389. 10.1021/acs.orglett.7b01230. 28640631

[ref33] Xia X. D.; Ren Y. L.; Chen J. R.; Yu X. L.; Lu L. Q.; Zou Y. Q.; Wan J.; Xiao W. J. Phototandem Catalysis: Efficient Synthesis of 3-Ester-3-hydroxy-2-oxindoles by a Visible Light-Induced Cyclization of Diazoamides through an Aerobic Oxidation Sequence. Chem.—Asian J. 2015, 10, 124–128. 10.1002/asia.201402990. 25294598

[ref34] Sagadevan A.; Charpe V. P.; Ragupathi A.; Hwang K. C. Visible Light Copper Photoredox-Catalyzed Aerobic Oxidative Coupling of Phenols and Terminal Alkynes: Regioselective Synthesis of Functionalized Ketones via C≡C Triple Bond Cleavage. J. Am. Chem. Soc. 2017, 139, 2896–2899. 10.1021/jacs.6b13113. 28177239

[ref35] Yi H.; Bian C. L.; Hu X.; Niu L. B.; Lei A. W. Visible light mediated efficient oxidative benzylic sp^3^ C–H to ketone derivatives obtained under mild conditions using O_2_. Chem. Commun. 2015, 51, 14046–14049. 10.1039/C5CC06015J. 26248184

[ref36] Ren L.; Yang M. M.; Tung C. H.; Wu L. Z.; Cong H. Visible-Light Photocatalysis Employing Dye-Sensitized Semiconductor: Selective Aerobic Oxidation of Benzyl Ethers. ACS Catal. 2017, 7, 8134–8138. 10.1021/acscatal.7b03029.

[ref37] Zou Y. Q.; Chen J. R.; Liu X. P.; Lu L. Q.; Davis R. L.; Jorgensen K. A.; Xiao W. J. Highly Efficient Aerobic Oxidative Hydroxylation of Arylboronic Acids: Photoredox Catalysis Using Visible Light. Angew. Chem., Int. Ed. 2012, 51, 784–788. 10.1002/anie.201107028. 22161996

[ref38] Pitre S. P.; McTiernan C. D.; Ismaili H.; Scaiano J. C. Mechanistic Insights and Kinetic Analysis for the Oxidative Hydroxylation of Arylboronic Acids by Visible Light Photoredox Catalysis: A Metal-Free Alternative. J. Am. Chem. Soc. 2013, 135, 13286–13289. 10.1021/ja406311g. 23952147

[ref39] Simlandy A. K.; Bhattacharyya B.; Pandey A.; Mukherjee S. Picosecond Electron Transfer from Quantum Dots Enables a General and Efficient Aerobic Oxidation of Boronic Acids. ACS Catal. 2018, 8, 5206–5211. 10.1021/acscatal.8b01078.

[ref40] Xiao Y. H.; Zhu C. M.; Liang R. B.; Huang Y. L.; Hai C. H.; Chen J. R.; Li M.; Zhong J. J.; Huang X. C. Building a cobaloxime-based metal-organic framework for photocatalytic aerobic oxidation of arylboronic acids to phenols. Chem. Commun. 2023, 59, 2239–2242. 10.1039/D2CC06945H. 36723203

[ref41] Johnson J. A.; Luo J.; Zhang X.; Chen Y. S.; Morton M. D.; Echeverría E.; Torres F. E.; Zhang J. Porphyrin-Metalation-Mediated Tuning of Photoredox Catalytic Properties in Metal-Organic Frameworks. ACS Catal. 2015, 5, 5283–5291. 10.1021/acscatal.5b00941.

[ref42] Toyao T.; Ueno N.; Miyahara K.; Matsui Y.; Kim T. H.; Horiuchi Y.; Ikeda H.; Matsuoka M. Visible-light, photoredox catalyzed, oxidative hydroxylation of arylboronic acids using a metal-organic framework containing tetrakis(carboxyphenyl)porphyrin groups. Chem. Commun. 2015, 51, 16103–16106. 10.1039/C5CC06163F. 26391908

[ref43] Paul A.; Chatterjee D.; Rajkamal; Halder T.; Banerjee S.; Yadav S. Metal free visible light photoredox activation of PhI(OAc)2 for the conversion of arylboronic acids to phenols. Tetrahedron Lett. 2015, 56, 2496–2499. 10.1016/j.tetlet.2015.03.107.

[ref44] Zhang G. Q.; Li Y.; Liu J. H. Acid-promoted metal-free protodeboronation of arylboronic acids. RSC Adv. 2017, 7, 34959–34962. 10.1039/C7RA05979E.

[ref45] Yu H. C.; Liu C.; Dai X. M.; Wang J.; Qiu J. S. Cyclometalated Ir(III) complexes-catalyzed aerobic hydroxylation of arylboronic acids induced by visible-light. Tetrahedron 2017, 73, 3031–3035. 10.1016/j.tet.2017.04.020.

[ref46] Xie H.-Y.; Han L.-S.; Huang S.; Lei X.; Cheng Y.; Zhao W.; Sun H.; Wen X.; Xu Q.-L. N-Substituted 3(10H)-Acridones as Visible-Light, Water-Soluble Photocatalysts: Aerobic Oxidative Hydroxylation of Arylboronic Acids. J. Org. Chem. 2017, 82, 5236–5241. 10.1021/acs.joc.7b00487. 28441486

[ref47] Wang Z. J.; Li R.; Landfester K.; Zhang K. A. I. Porous conjugated polymer via metal-free synthesis for visible light-promoted oxidative hydroxylation of arylboronic acids. Polymer 2017, 126, 291–295. 10.1016/j.polymer.2017.04.052.

[ref48] Fang Y. D.; Zhao R.; Yao Y.; Liu Y.; Chang D. H.; Yao M.; Shi L. Trichloroacetonitrile as an efficient activating agent for the ipso-hydroxylation of arylboronic acids to phenolic compounds. Org. Biomol. Chem. 2019, 17, 7558–7563. 10.1039/C9OB01568J. 31373339

[ref49] Begum T.; Gogoi A.; Gogoi P. K.; Bora U. Catalysis by mont K-10 supported silver nanoparticles: a rapid and green protocol for the efficient ipso-hydroxylation of arylboronic acids. Tetrahedron Lett. 2015, 56, 95–97. 10.1016/j.tetlet.2014.11.018.

[ref50] Dar B. A.; Bhatti P.; Singh A. P.; Lazar A.; Sharma P. R.; Sharma M.; Singh B. Clay entrapped Cu(OH)x as an efficient heterogeneous catalyst for ipso-hydroxylation of arylboronic acids. Appl. Catal. A-Gen. 2013, 466, 60–67. 10.1016/j.apcata.2013.06.051.

[ref51] Gogoi N.; Gogoi P. K.; Borah G.; Bora U. Grafting of Ru(III) complex onto nanosilica and its implication as heterogeneous catalyst for aerobic oxidative hydroxylation of arylboronic acids. Tetrahedron Lett. 2016, 57, 4050–4052. 10.1016/j.tetlet.2016.07.070.

[ref52] Inamoto K.; Nozawa K.; Yonemoto M.; Kondo Y. Micellar system in copper-catalysed hydroxylation of arylboronic acids: facile access to phenols. Chem. Commun. 2011, 47, 11775–11777. 10.1039/c1cc14974a. 21959336

[ref53] Zheng J.; Lin S. Y.; Zhu X. H.; Jiang B. W.; Yang Z.; Pan Z. Y. Reductant-directed formation of PS-PAMAM-supported gold nanoparticles for use as highly active and recyclable catalysts for the aerobic oxidation of alcohols and the homocoupling of phenylboronic acids. Chem. Commun. 2012, 48, 6235–6237. 10.1039/c2cc31948a. 22595867

[ref54] Wang L.; Zhang W.; Sheng Su D.; Meng X. J.; Xiao F. S. Supported Au nanoparticles as efficient catalysts for aerobic homocoupling of phenylboronic acid. Chem. Commun. 2012, 48, 5476–5478. 10.1039/c2cc31115a. 22543590

[ref55] Weng W. Z.; Liang H.; Zhang B. Visible-Light-Mediated Aerobic Oxidation of Organoboron Compounds Using in Situ Generated Hydrogen Peroxide. Org. Lett. 2018, 20, 4979–4983. 10.1021/acs.orglett.8b02095. 30091613

[ref56] Das S. K.; Bhattacharjee P.; Bora U. Ascorbic Acid as a Highly Efficient Organocatalyst for ipso-Hydroxylation of Arylboronic Acid. ChemistrySelect 2018, 3, 2131–2134. 10.1002/slct.201703036.

[ref57] Molloy J. J.; Clohessy T. A.; Irving C.; Anderson N. A.; Lloyd-Jones G. C.; Watson A. J. B. Chemoselective oxidation of aryl organoboron systems enabled by boronic acid-selective phase transfer. Chem. Sci. 2017, 8, 1551–1559. 10.1039/C6SC04014D. 28572912 PMC5452267

[ref58] Zhu C.; Wang R.; Falck J. R. Mild and Rapid Hydroxylation of Aryl/Heteroaryl Boronic Acids and Boronate Esters with N-Oxides. Org. Lett. 2012, 14, 3494–3497. 10.1021/ol301463c. 22731862 PMC3391750

[ref59] Saikia I.; Hazarika M.; Hussian N.; Das M. R.; Tamuly C. Biogenic synthesis of Fe_2_O_3_@SiO_2_ nanoparticles for ipso-hydroxylation of boronic acid in water. Tetrahedron Lett. 2017, 58, 4255–4259. 10.1016/j.tetlet.2017.09.075.

[ref60] Chatterjee N.; Chowdhury H.; Sneh K.; Goswami A. Hydroxylation of aryl- and alkylboronic acids/esters mediated by iodobenzene diacetate-an avenue for using organoboronic acids/esters as nucleophiles for hydroxylation reactions. Tetrahedron Lett. 2015, 56, 172–174. 10.1016/j.tetlet.2014.11.058.

[ref61] Gupta S.; Chaudhary P.; Srivastava V.; Kandasamy J. A chemoselective ipso-hydroxylation of arylboronic acids using urea-hydrogen peroxide under catalyst free condition. Tetrahedron Lett. 2016, 57, 2506–2510. 10.1016/j.tetlet.2016.04.099.

[ref62] Molloy J. J.; Watson A. Chemoselective oxidation of aryl organoboron systems enabled by boronic acid-selective phase transfer. Abstr. Pap. Am. Chem. Soc. 2017, 254, 1. 10.1039/c6sc04014d PMC5452267 28572912

[ref63] Gogoi A.; Bora U. A mild and efficient protocol for the ipso-hydroxylation of arylboronic acids. Tetrahedron Lett. 2013, 54, 1821–1823. 10.1016/j.tetlet.2013.01.079.

[ref64] Huang J. M.; Chen D. S. A Mild and Highly Efficient Conversion of Arylboronic Acids into Phenols by Oxidation with MCPBA. Synlett 2013, 24, 499–501. 10.1055/s-0032-1318197.

[ref65] Chatterjee N.; Goswami A. Organic hypervalent iodine (III) catalyzed ipso-hydroxylation of aryl- and alkylboronic acids/esters. Tetrahedron Lett. 2015, 56, 1524–1527. 10.1016/j.tetlet.2015.01.118.

[ref66] Elumalai V.; Hansen J. H. A scalable and green one-minute synthesis of substituted phenols. RSC Adv. 2020, 10, 40582–40587. 10.1039/D0RA08580D. 35520826 PMC9057563

[ref67] Ceballos J.; Garreau M.; Waser J. An Alternative One-Electron Oxidation Strategy to Access Hypervalent Iodine Reagents. Chem 2019, 5, 2287–2289. 10.1016/j.chempr.2019.08.005.

[ref68] Wengryniuk S. E. More Than Just Acetates: PhI(OAc)^2^ Enables C–H Halogenation of Arenes. Chem 2019, 5, 258–260. 10.1016/j.chempr.2019.01.009.

