# Supplementary material for: Scalable and Practical Approach of Phenol Formation from Hydroxylation of Arylboronic Acids under Metal-, Photocatalyst-, and Light-Free Conditions
Source: ACS Omega. 2025 Jan 13;10(3):2421–7. doi: 10.1021/acsomega.4c02844 (PMC11780564; doi:10.1021/acsomega.4c02844)
Supplement: Supplementary file 1 — ao4c02844_si_001.pdf [file ao4c02844_si_001.pdf]

Electronic Supporting Information for:

Scalable and Practical Approach of Phenol Formation from Hydroxylation of Arylboronic Acids under Metal-, Photocatalyst-, and Light-Free Conditions

Shuqing Cai, [a] *Noreen Rehmat,* [a] *Zafar Mahmood*,*[a] *Qian Chen,*[a] *Yanping Huo,*[a,b] *Shaomin Ji*,*[a,b]

[a] School of Chemical Engineering and Light Industry, Guangdong University of Technology, Guangzhou 510006, P.R. China. E-mail: smji@gdut.edu.cn

[b] Guangdong Provincial Laboratory of Chemistry and Fine Chemical Engineering Jieyang Center, Jieyang 515200, P.R. China

Contents

1. General Information..Page S3
2. General Procedure for Synthesis of Phenols and Characterization Data…..................Page S4
3. Optimization of Reaction Conditions and Controlled Experiments……………........Page S11
4. Scaled-up Raection and Electron paramagnetic resonance Study...............................Page S12
5. NMR Characterization Data of Isolated Product..Page S14

1. General information

All reagents were purchased from Sigma-Aldrich, and used without further purification. Reactions were monitored by thin layer chromatography (TLC) and visualized by UV lamp (256 nm). Flash column chromatography was performed using 230-400 mesh silica gel. Yields refer to purified compounds unless otherwise noted. ^{1}H NMR (400 MHz), ^{13}C NMR (101 MHz) spectra were obtained on Bruker 400M nuclear resonance spectrometers. ^{1}H NMR and ^{13}C NMR chemical shifts are referenced with respect to $CDCl_3$ (^{1}H NMR: residual $CDCl_3$ at δ 7.26, ^{13}C NMR: $CDCl_3$ triplet at δ 77.16) and DMSO-d_6 (^{1}H NMR: residual DMSO-d_6 at δ 2.50, ^{13}C NMR: DMSO-d_6 quintet at δ 39.50) Data for ^{1}H NMR spectra were reported as chemical shifts (δ ppm), broad peak (b), multiplicity (s = singlet, d = doublet, t = triplet, q = quartet, m = multiplet, dd = doublet of doublet, dt = doublet of triplet, etc.), coupling constant (Hz) and integration; data for ^{13}C NMR were reported in terms of chemical shift (δ ppm) and no special nomenclature is used for equivalent carbons.

2. General Procedure for Synthesis of Phenols and Characterization Data

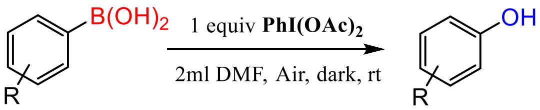

Scheme S1 Synthesis of phenols

An oven-dried 20 mL quartz test tube equipped with a magnetic stir bar was charged with 4-Formylphenylboronic acid (45 mg, 0.3 mmol, 1.0 equiv.), $PhI(OAc)_2$ (96.6mg, 0.3mmol, 1.0equiv) and 2mL DMF was added in tube. Then cover was closed and Aluminum foil is used to protect the tube from light. The resulting mixture was stirred for 2 hours. After the mentioned time, the water (10.0 mL) was added to quench the reaction, the organic layer was separated, and the aqueous layer was extracted with EtOAc (3 × 5.0 mL). The combined organic layers were dried over Na_2SO_4, filtered, and the filtrate was concentrated under reduced pressure to dryness. The residue was then purified by flash chromatography on silica gel (EtOAc/PE = 1/2) directly to give the desired product (96% yield, Faint yellow solid).

4-Hydroxybenzaldehyde (1b)

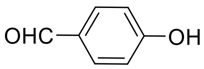

^{1}H NMR (400 MHz, DMSO-d_6) δ 10.59 (s, 1H), 9.79 (s, 1H), 7.76 (d, J = 8.6 Hz, 2H), 6.93 (d, J = 8.5 Hz, 2H). ^{13}C NMR (101 MHz, DMSO) δ 191.41, 163.78, 132.57, 128.91, 116.31.

4-Methoxyphenol (2b)

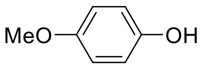

^{1}H NMR (400 MHz, Chloroform-d) δ 6.98 – 6.59 (m, 4H), 4.87 (s, 1H), 3.77 (s, 3H). ^{13}C NMR (101 MHz, $CDCl_3$) δ 153.67, 149.53, 116.13, 114.96, 55.89.

4-Fluorophenol (3b)

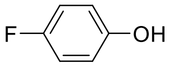

^{1}H NMR (400 MHz, Chloroform-*d*) δ 7.24 – 7.15 (m, 2H), 6.81 – 6.71 (m, 2H), 4.60 (s, 1H).

^{13}C NMR (101 MHz, $CDCl_3$) δ 154.05, 129.58, 125.71, 116.73.

5-4-Chlorophenol (4b)

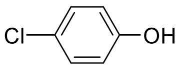

^{1}H NMR (400 MHz, Chloroform-*d*) δ 7.24 – 7.15 (m, 2H), 6.81 – 6.74 (m, 2H), 5.03 (s, 1H).

^{13}C NMR (101 MHz, $CDCl_3$) δ 153.95, 129.60, 125.83, 116.72, 116.37, 77.39, 77.08, 76.76.

4-Bromophenol (5b)

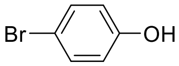

^{1}H NMR (400 MHz, DMSO-d_6) δ 9.69 (s, 1H), 7.35 – 7.27 (m, 2H), 6.76 – 6.69 (m, 2H).

^{13}C NMR (101 MHz, DMSO) δ 157.22, 132.48, 118.00, 110.36.

5-Iodophenol (6b)

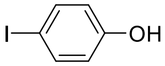

^{1}H NMR (400 MHz, Chloroform-*d*) δ 7.56 – 7.47 (m, 2H), 6.67 – 6.58 (m, 2H), 5.07 (s, 1H).

^{13}C NMR (101 MHz, $CDCl_3$) δ 155.42, 138.46, 117.83, 82.65.

4-(Trifluoromethyl)phenol (7b)

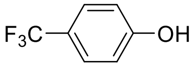

^{1}H NMR (400 MHz, Chloroform-*d*) δ 7.49 (d, *J* = 8.6 Hz, 2H), 6.91 (d, *J* = 8.5 Hz, 2H), 6.62 (s, 1H). ^{13}C NMR (101 MHz, $CDCl_3$) δ 158.31, 128.47, 127.25, 127.21, 127.17, 127.13, 125.78, 123.53, 123.21, 123.09, 122.88, 122.56, 120.39, 115.49.

4-Cyanophenol (8b)

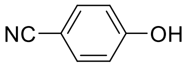

^{1}H NMR (400 MHz, Chloroform-*d*) δ 7.61 – 7.51 (m, 2H), 6.97 – 6.90 (m, 2H), 6.51 (s, 1H). ^{13}C NMR (101 MHz, $CDCl_3$) δ 160.11, 134.35, 119.25, 116.47, 103.22.

4-Nitrophenol (9b)

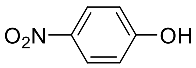

^{1}H NMR (400 MHz, Chloroform-*d*) δ 8.23 – 8.13 (m, 2H), 6.97 – 6.89 (m, 2H), 6.52 (s, 1H). ^{13}C NMR (101 MHz, $CDCl_3$) δ 161.66, 141.49, 126.34, 115.77.

3-Methoxyphenol (10b)

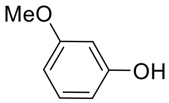

^{1}H NMR (400 MHz, Chloroform-*d*) δ 7.14 (t, *J* = 8.2 Hz, 1H), 6.51 (ddd, *J* = 8.3, 2.3, 0.9 Hz, 1H), 6.47 – 6.42 (m, 2H), 4.65 (s, 1H), 3.78 (s, 3H). ^{13}C NMR (101 MHz, $CDCl_3$) δ 160.85, 156.79, 130.23, 107.99, 106.44, 101.64, 55.34.

2-Methoxyphenol (11b)

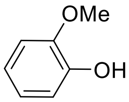

^{1}H NMR (400 MHz, Chloroform-*d*) δ 6.97 – 6.93 (m, 1H), 6.92 – 6.85 (m, 3H), 5.68 (s, 1H), 3.89 (s, 3H). ^{13}C NMR (101 MHz, $CDCl_3$) δ 146.61, 145.70, 121.48, 120.17, 114.57, 110.76, 55.89.

3,5-Dimethoxyphenol (12b)

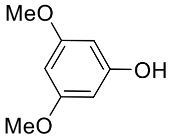

^{1}H NMR (400 MHz, Chloroform-*d*) δ 6.05 (dd, *J* = 16.4, 2.2 Hz, 3H), 5.43 (s, 0H), 3.75 (s, 7H). ^{13}C NMR (101 MHz, $CDCl_3$) δ 161.62, 157.45, 94.31, 93.18, 55.37.

2,6-Dimethoxyphenol (13b)

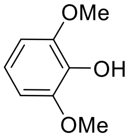

^{1}H NMR (400 MHz, Chloroform-*d*) δ 6.80 (dd, *J* = 8.7, 8.0 Hz, 1H), 6.58 (d, *J* = 8.3 Hz, 2H), 5.52 (s, 1H), 3.89 (s, 6H). ^{13}C NMR (101 MHz, $CDCl_3$) δ 147.27, 134.88, 119.09, 104.92, 56.28.

2,5-Dimethoxyphenol (14b)

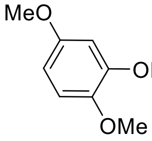

^{1}H NMR (400 MHz, Chloroform-*d*) δ 6.77 (d, *J* = 8.9 Hz, 1H), 6.56 (d, *J* = 2.9 Hz, 1H), 6.37 (dd, *J* = 8.8, 2.9 Hz, 1H), 5.69 (s, 1H), 3.84 (s, 3H), 3.74 (s, 3H). ^{13}C NMR (101 MHz, $CDCl_3$) δ 154.58, 146.45, 140.99, 111.51, 104.26, 101.77, 56.59, 55.67.

3,4-Dimethoxyphenol (15b)

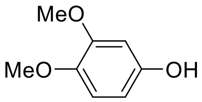

^{1}H NMR (400 MHz, Chloroform-*d*) δ 6.73 (d, *J* = 8.6 Hz, 1H), 6.47 (d, *J* = 2.8 Hz, 1H), 6.34 (dd, *J* = 8.6, 2.8 Hz, 1H), 3.83 (d, *J* = 6.1 Hz, 6H). ^{13}C NMR (101 MHz, $CDCl_3$) δ 150.04, 149.96, 143.29, 112.34, 105.77, 100.59, 56.56, 55.84.

2,4,6-Trimethoxyphenol (16b)

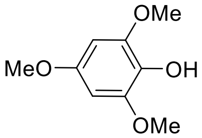

^{1}H NMR (400 MHz, Chloroform-*d*) δ 6.80 (s, 2H), 4.45 (s, 1H), 2.22 (s, 9H). ^{13}C NMR (101 MHz, $CDCl_3$) δ 149.89, 129.29, 129.12, 122.79, 20.40, 15.83.

Naphthalen-1-ol (17b)

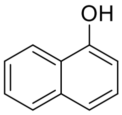

^{1}H NMR (400 MHz, Chloroform-*d*) δ 8.25 – 8.18 (m, 1H), 7.90 – 7.81 (m, 1H), 7.59 – 7.44 (m, 3H), 7.33 (t, *J* = 7.8 Hz, 1H), 6.82 (dd, *J* = 7.4, 1.0 Hz, 1H), 5.93 – 4.90 (m, 1H). ^{13}C NMR (101 MHz, $CDCl_3$) δ 151.37, 134.82, 127.76, 126.52, 125.91, 125.36, 124.41, 121.58, 120.79, 108.74.

Naphthalen-2-ol (18b)

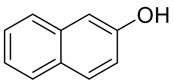

^{1}H NMR (400 MHz, DMSO-d_6) δ 10.10 (s, 1H), 8.18 – 8.09 (m, 1H), 7.80 (d, *J* = 6.8 Hz, 1H), 7.49 – 7.40 (m, 2H), 7.36 – 7.26 (m, 2H), 6.88 (dd, *J* = 7.0, 1.6 Hz, 1H). ^{13}C NMR (101 MHz, DMSO) δ 153.62, 134.88, 127.83, 126.88, 126.53, 125.03, 124.99, 122.44, 118.78, 108.49.

Anthracen-2-ol (20b)

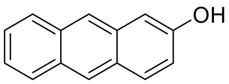

^{1}H NMR (400 MHz, DMSO-d_6) δ 9.89 (s, 1H), 8.43 (s, 1H), 8.26 (s, 1H), 8.00 – 7.93 (m, 4H), 7.46 – 7.35 (m, 1H), 7.23 (d, *J* = 2.3 Hz, 1H), 7.16 (dd, *J* = 9.0, 2.4 Hz, 1H). ^{13}C NMR (101

MHz, DMSO) δ 154.83, 132.86, 131.68, 129.85, 129.34, 128.12, 127.32, 125.99, 125.46, 124.07, 122.86, 120.60, 106.46.

4-Phenylphenol (21b)

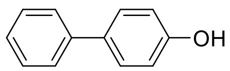

^{1}H NMR (400 MHz, DMSO-d_6) δ 9.54 (s, 1H), 7.56 (d, *J* = 7.0 Hz, 2H), 7.48 (d, *J* = 8.6 Hz, 2H), 7.40 (t, *J* = 7.7 Hz, 2H), 7.27 (t, *J* = 7.3 Hz, 1H), 6.85 (d, *J* = 8.6 Hz, 2H). ^{13}C NMR (101 MHz, DMSO) δ 157.59, 140.70, 131.40, 129.26, 128.20, 126.82, 126.42, 116.19.

3-Phenylphenol (22b)

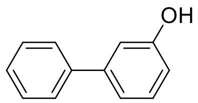

1H NMR (400 MHz, Chloroform-d) δ 7.54 – 7.51 (m, 3H), 7.40 – 7.36 (m, 2H), 7.33 – 7.26 (m, 1H), 7.12 (d, J = 9.4 Hz, 1H), 7.03 – 7.01 (m, 1H), 6.83 – 6.71 (m, 1H). ^{13}C NMR (101 MHz, $CDCl_3$) δ 155.88, 143.04, 140.76, 130.03, 128.79, 127.52, 127.15, 119.80, 114.25, 114.15.

4-(2-Thienyl)Phenol (23b)

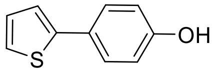

^{1}H NMR (400 MHz, Chloroform-*d*) δ 7.49 (d, *J* = 8.6 Hz, 2H), 7.21 (dd, *J* = 5.1, 1.2 Hz, 1H), 7.19 (dd, *J* = 3.6, 1.2 Hz, 1H), 7.05 (dd, *J* = 5.1, 3.6 Hz, 1H), 6.87 – 6.84 (m, 3H), 5.07 (s, 1H). ^{13}C NMR (101 MHz, $CDCl_3$) δ 155.24, 144.28, 127.93, 127.48, 127.46, 123.86, 122.11, 115.77.

4-(Naphthalen-2-yl)phenol (26b)

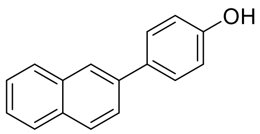

^{1}H NMR (400 MHz, DMSO-d_6) δ 9.62 (s, 1H), 8.11 (d, J = 1.8 Hz, 1H), 7.98 – 7.87 (m, 3H), 7.79 (dd, J = 8.6, 1.9 Hz, 1H), 7.70 – 7.60 (m, 2H), 7.50 (dqd, J = 8.1, 6.9, 1.5 Hz, 2H), 6.96 – 6.88 (m, 2H). ^{13}C NMR (101 MHz, DMSO) δ 157.76, 138.04, 133.94, 132.21, 131.14, 128.74, 128.53, 128.42, 127.90, 126.72, 126.05, 125.35, 124.38, 116.32.

Hydroxypyrene (27b)

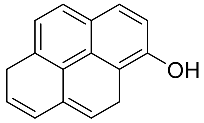

^{1}H NMR (400 MHz, DMSO-d_6) δ 10.63 (s, 1H), 8.34 (d, J = 9.1 Hz, 1H), 8.14 – 8.09 (m, 3H), 8.02 (dd, J = 11.3, 9.1 Hz, 2H), 7.99 – 7.94 (m, 1H), 7.89 (d, J = 8.9 Hz, 1H), 7.60 (d, J = 8.3 Hz, 1H). ^{13}C NMR (101 MHz, DMSO) δ 152.16, 131.38, 131.34, 127.41, 126.19, 126.11, 125.50, 125.44, 124.46, 123.90, 123.79, 123.62, 123.60, 121.42, 118.08, 113.24.

N-(4-hydroxyphenyl)carbazole (28b)

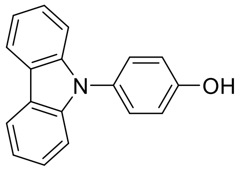

^{1}H NMR (400 MHz, DMSO-d_6) δ 9.87 (s, 1H), 8.22 (d, J = 7.7 Hz, 2H), 7.45 – 7.32 (m, 4H), 7.32 – 7.21 (m, 4H), 7.05 (d, J = 8.6 Hz, 2H). ^{13}C NMR (101 MHz, DMSO) δ 157.43, 141.21, 128.70, 128.23, 126.52, 122.82, 120.86, 120.05, 117.04, 110.01.

9-Phenyl-9H-carbazol-2-ol (29b)

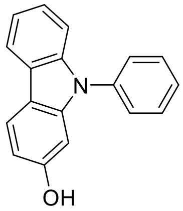

^{1}H NMR (400 MHz, DMSO-d_6) δ 9.56 (s, 1H), 8.03 (dd, J = 24.8, 8.5 Hz, 2H), 7.68 (t, J = 7.7 Hz, 2H), 7.63 – 7.47 (m, 3H), 7.29 (d, J = 6.0 Hz, 2H), 7.21 (ddd, J = 8.0, 5.8, 2.3 Hz, 1H), 6.80 – 6.72 (m, 2H). ^{13}C NMR (101 MHz, DMSO) δ 157.51, 142.33, 140.55, 137.52, 130.61, 128.04, 127.12, 124.84, 123.80, 121.80, 120.38, 119.73, 115.69, 110.11, 109.60, 95.74.

3. Optimization of Reaction Conditions and Controlled Experiments

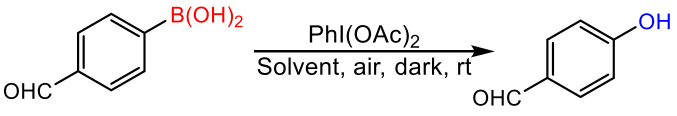

Table S1. Optimization of the Reaction Conditions[a]

Entry	Light source	Oxidant	Solvent	Yield of 2a (%)
1	Blue LED	1 equiv $PhI(OAc)_2$	DMF	74
2	Green LED	1 equiv $PhI(OAc)_2$	DMF	86
3	Red LED	1 equiv $PhI(OAc)_2$	DMF	95
4	Dark	1 equiv $PhI(OAc)_2$	DMF	96
5	Dark	1 equiv $PhI(OAc)_2$	ACN	35
6	Dark	1 equiv $PhI(OAc)_2$	THF	68
7	Dark	1 equiv $PhI(OAc)_2$	DMSO	84
8	Dark	0.5 equiv $PhI(OAc)_2$	DMF	62
9	Dark	0.25 equiv $PhI(OAc)_2$	DMF	20
10	Dark	0	DMF	0
11[b]	Dark	1 equiv $PhI(OAc)_2$	DMF	50

[a] Reaction conditions: **1a** (0.2 mmol), anhydrous solvent (2 mL), air atmosphere, in the dark, 2h, isolated yields. [b] Under the N_2 atmosphere.

Table S2. Controlled Experiments and Radical Capturing Study[a]

Entry	Light Source	Atmosphere	Radical capturing agent	Product state
1	Blue LED	Air	–[b]	–[c]
2	Green LED	Air	–[b]	–[c]
3	Red LED	Air	–[b]	–[c]
4	Dark	Air	–[b]	–[c]
5	Dark	Air	–[b]	+
6	Dark	N_2	–[b]	+
7	Dark	Air	Tempo	+
8	Dark	Air	1,4-Benzoquinone	+

[a] Conditions: **1a** (0.2 mmol), anhydrous solvent (2 mL), air atmosphere, in the dark. [b] Not applicable. [C] No product

4. Scaled-up reaction and Electron paramagnetic resonance Study

An oven-dried 50 mL round-bottom flask equipped with a magnetic stir bar was charged with ten folds large amount of 4-Formylphenylboronic acid (450 mg, 3 mmol, 10.0 equiv.) compared to optimized conditions, and $PhI(OAc)_2$ (966mg, 3mmol, 10.0equiv), 20mL DMF were added in tube. Then the flask was closed with a rubber cork and protect it from light using Aluminum foil. The resulting mixture was stirred overnight and desired product was obtained without any significant decrease in the yield.

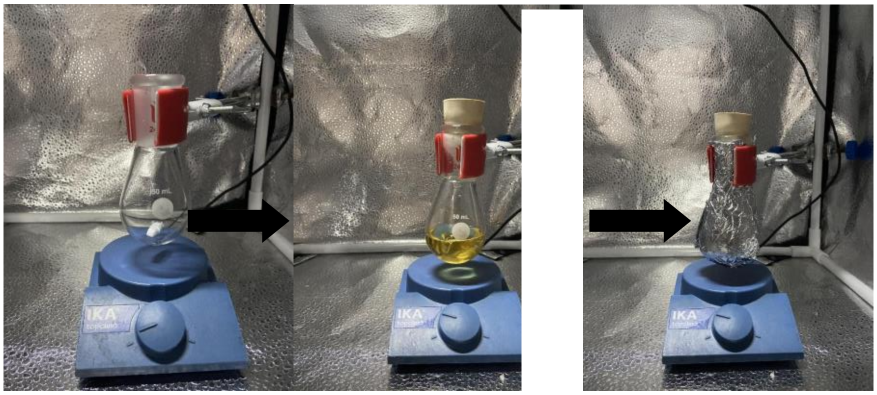

Figure S1. Scaled-up reaction setup

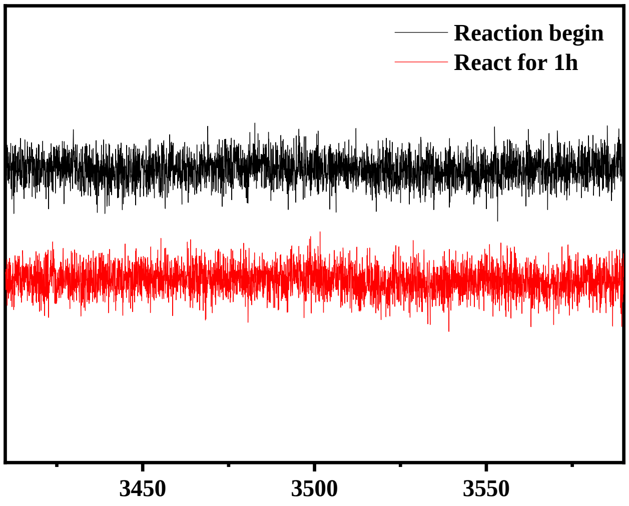

Figure S2. EPR Spectrum of the 4-Formylphenylboronic acid and $PhI(OAc)_2$ (0.2 mmol:0.2 mmol) in DMF, monitored from the reaction begin (black line) and after 1h reaction (red line).

5. NMR Characterization Data of the Isolated Product

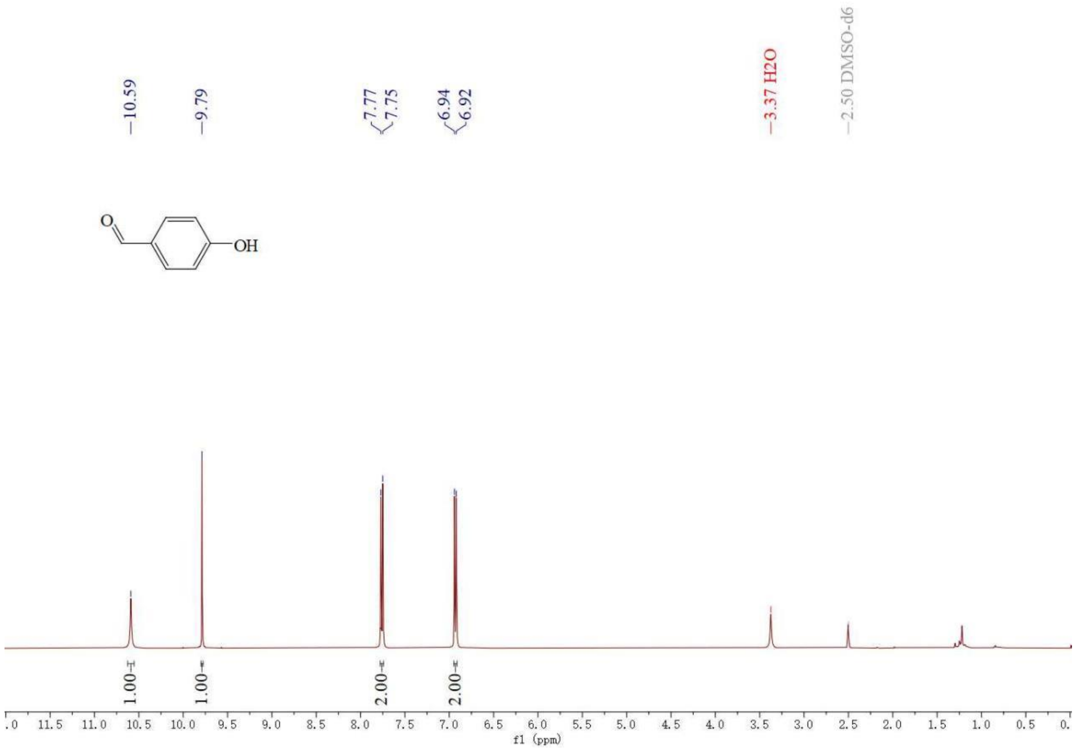

Figure S3. ^{1}HNMR spectrum of isolated product **1b** (400 MHz, DMSO-d$_6$)

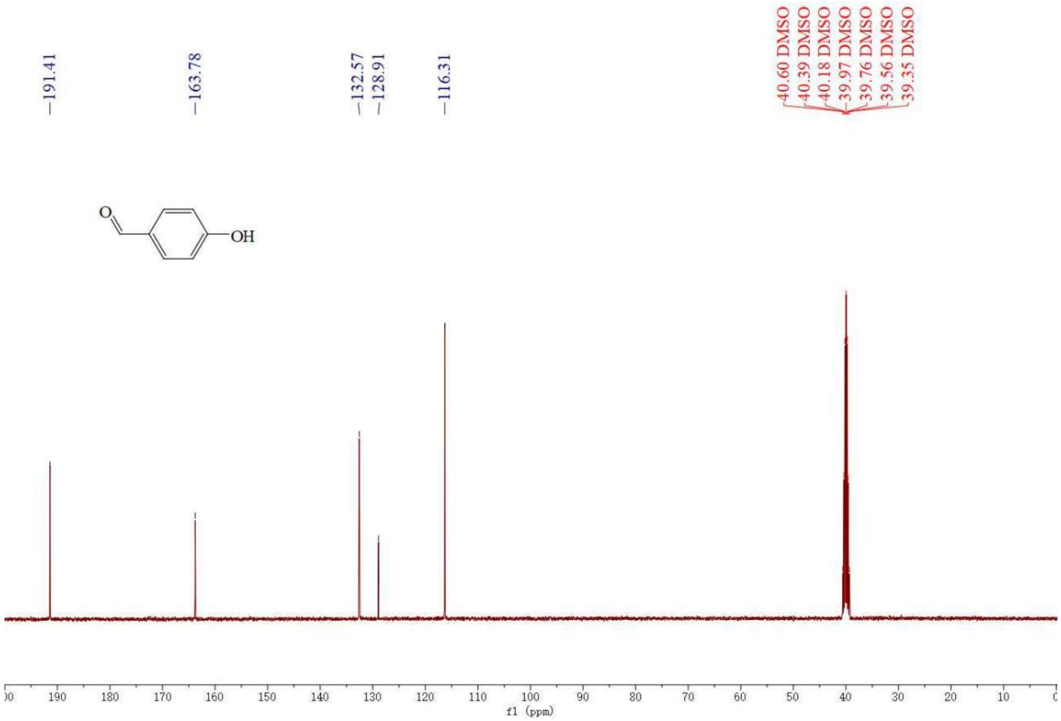

Figure S4. ^{13}CNMR spectrum of isolated product **1b** (100 MHz, DMSO-d$_6$)

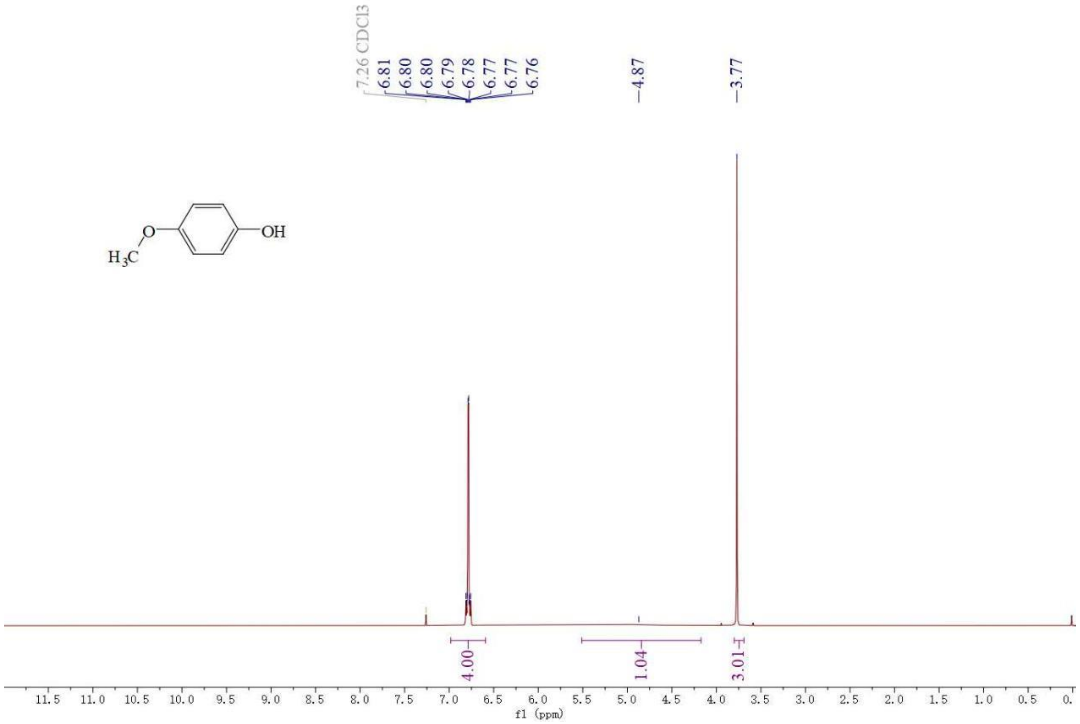

Figure S5. ^{1}HNMR spectrum of isolated product **2b** (400 MHz, chloroform-*d*)

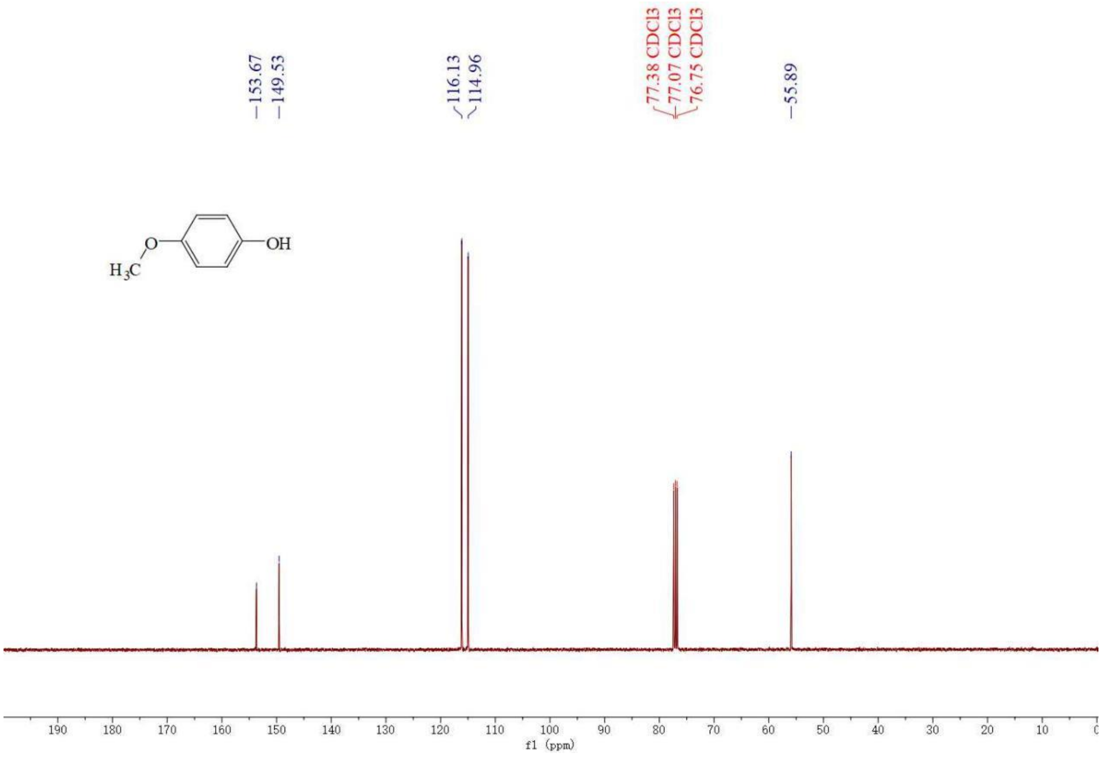

Figure S6. ^{13}CNMR spectrum of isolated product **2b** (100 MHz, chloroform-*d*)

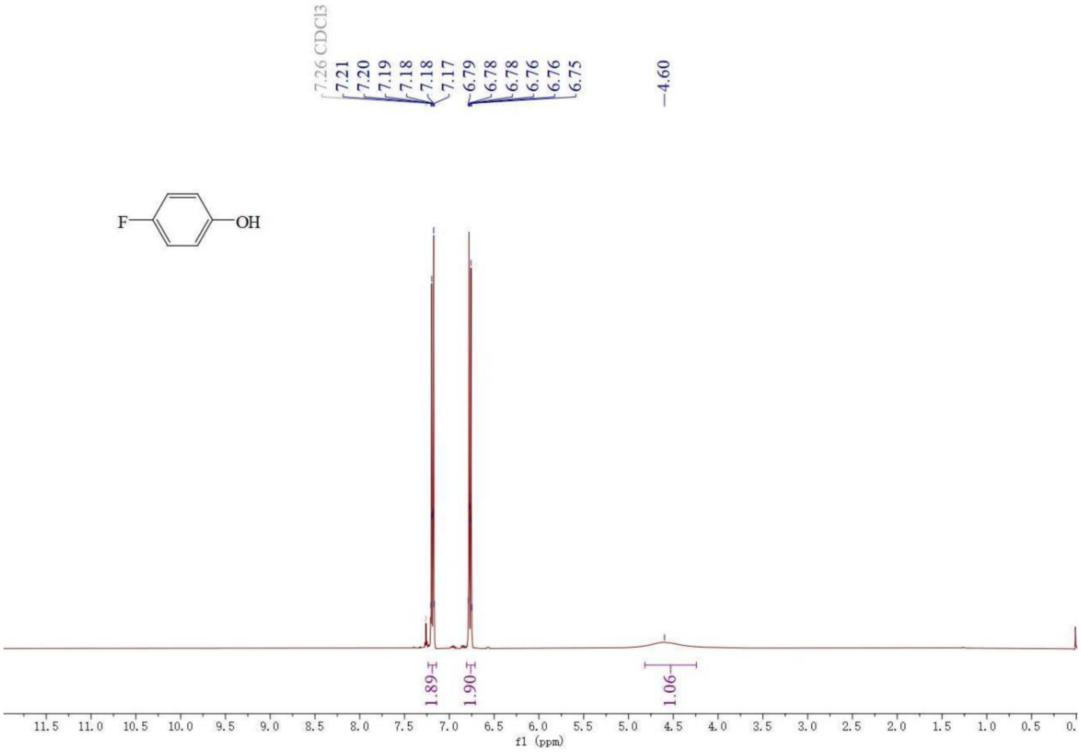

Figure S7. ^{1}HNMR spectrum of isolated product **3b** (400 MHz, chloroform-*d*)

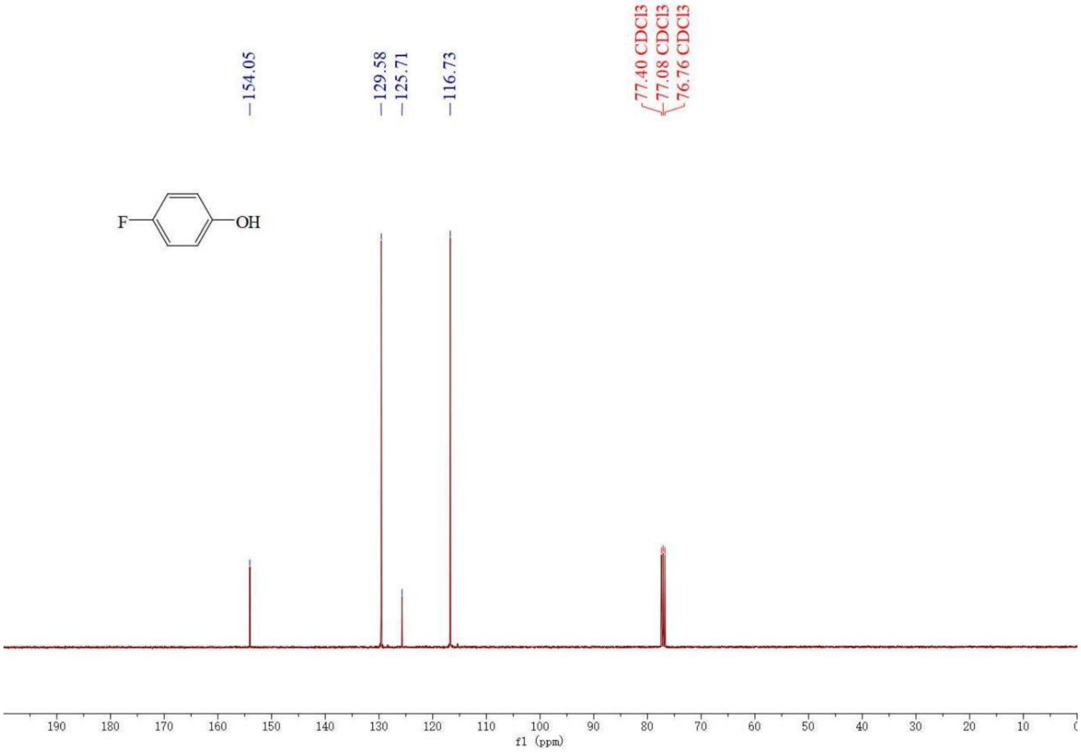

Figure S8. ^{13}CNMR spectrum of isolated product **3b** (100 MHz, chloroform-*d*)

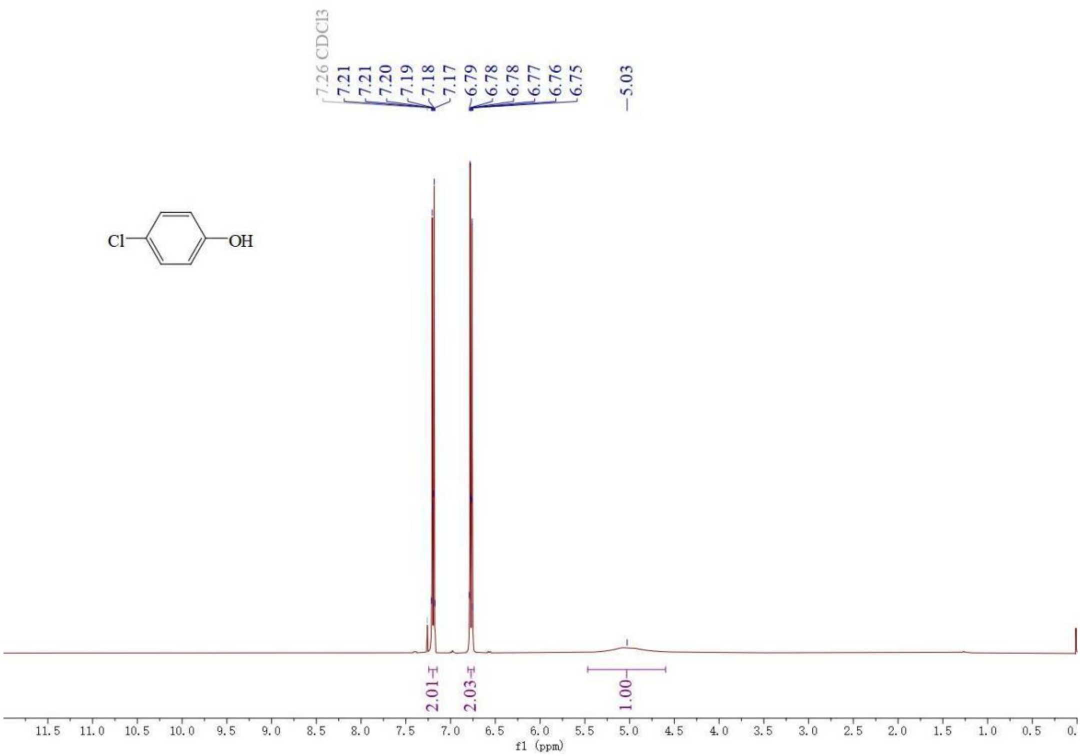

Figure S9. ^{1}HNMR spectrum of isolated product **4b** (400 MHz, chloroform-*d*)

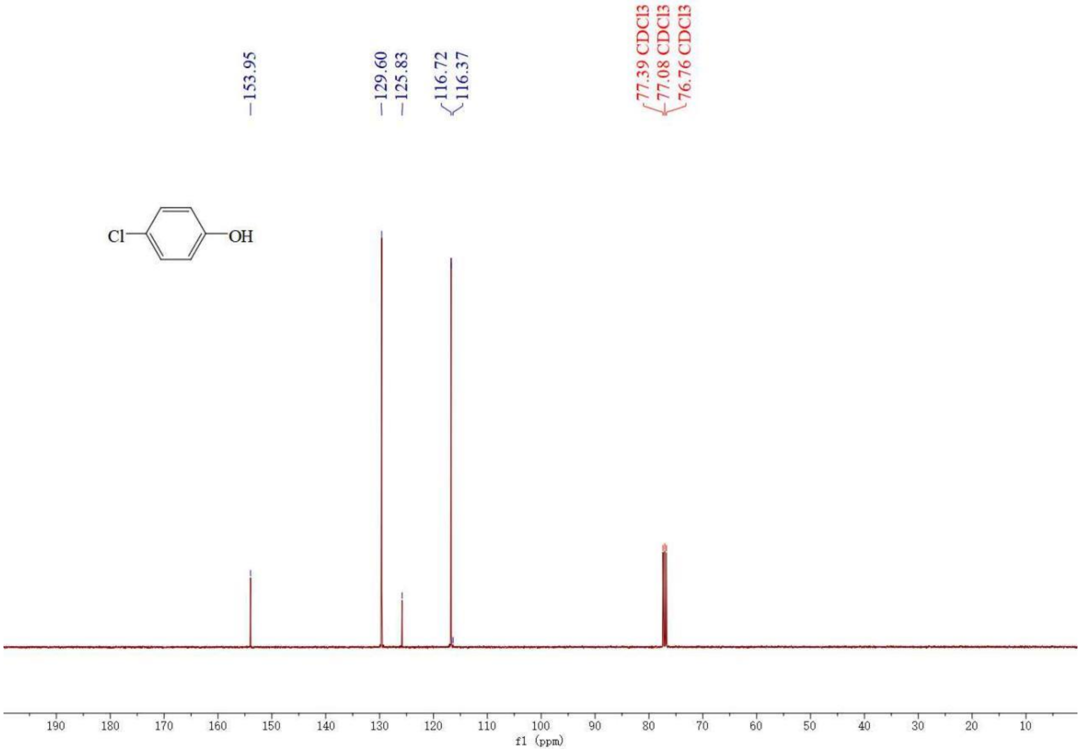

Figure S10. ^{13}CNMR spectrum of isolated product **4b** (100 MHz, chloroform-*d*)

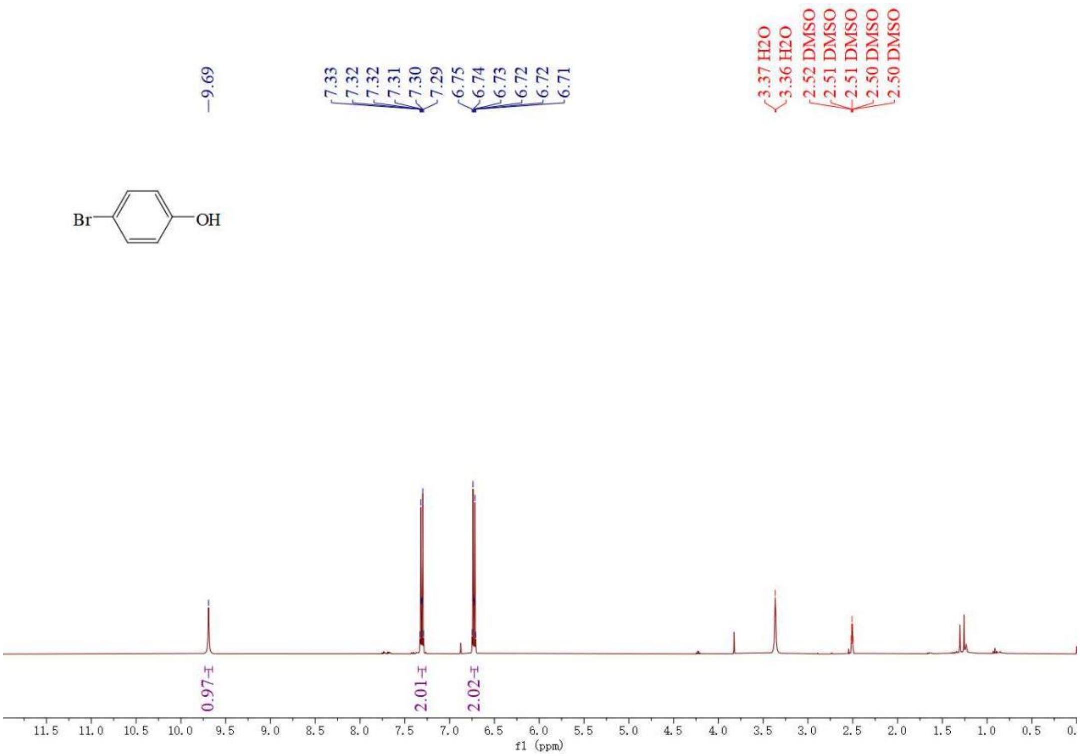

Figure S11. ^{1}HNMR spectrum of isolated product **5b** (400 MHz, DMSO-d_6)

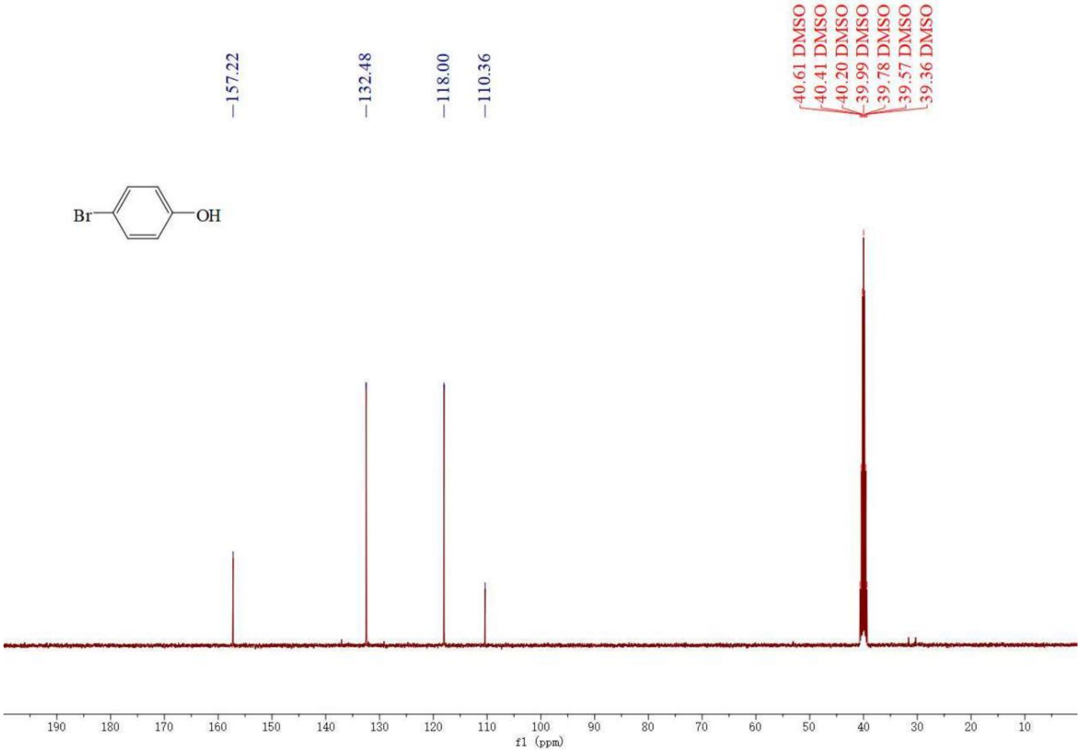

Figure S12. ^{13}CNMR spectrum of isolated product **5b** (100 MHz, DMSO-d_6)

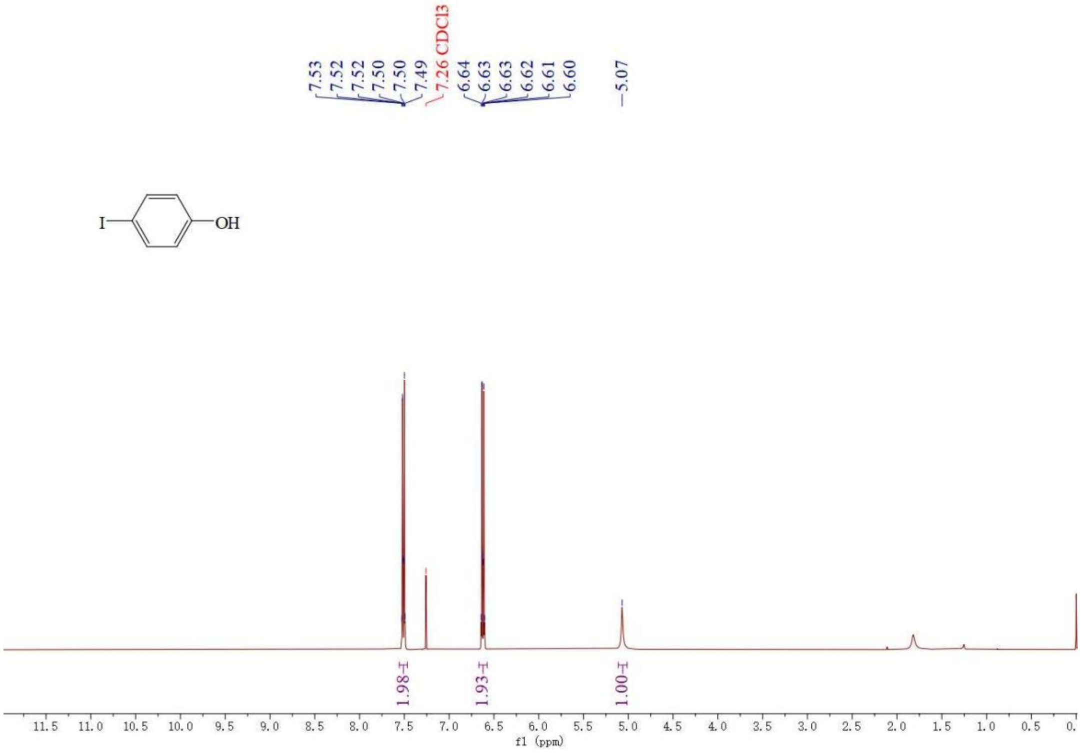

Figure S13. ^{1}HNMR spectrum of isolated product **6b** (400 MHz, chloroform-*d*)

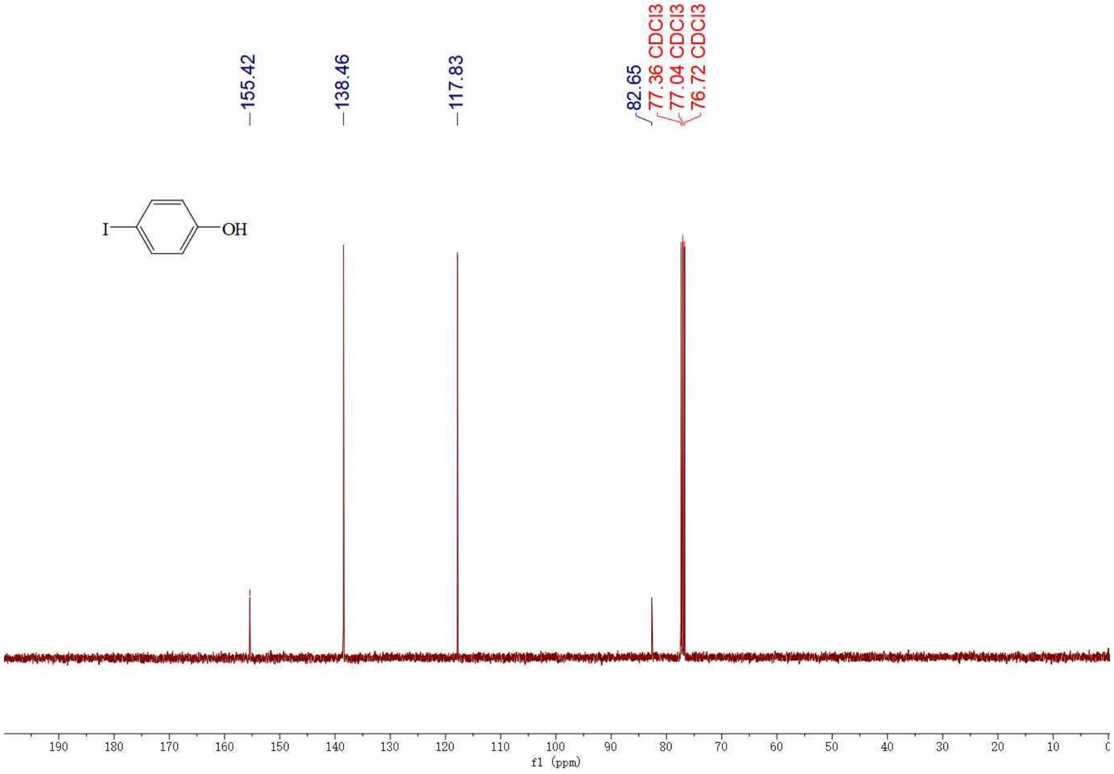

Figure S14. ^{13}CNMR spectrum of isolated product **6b** (100 MHz, chloroform-*d*)

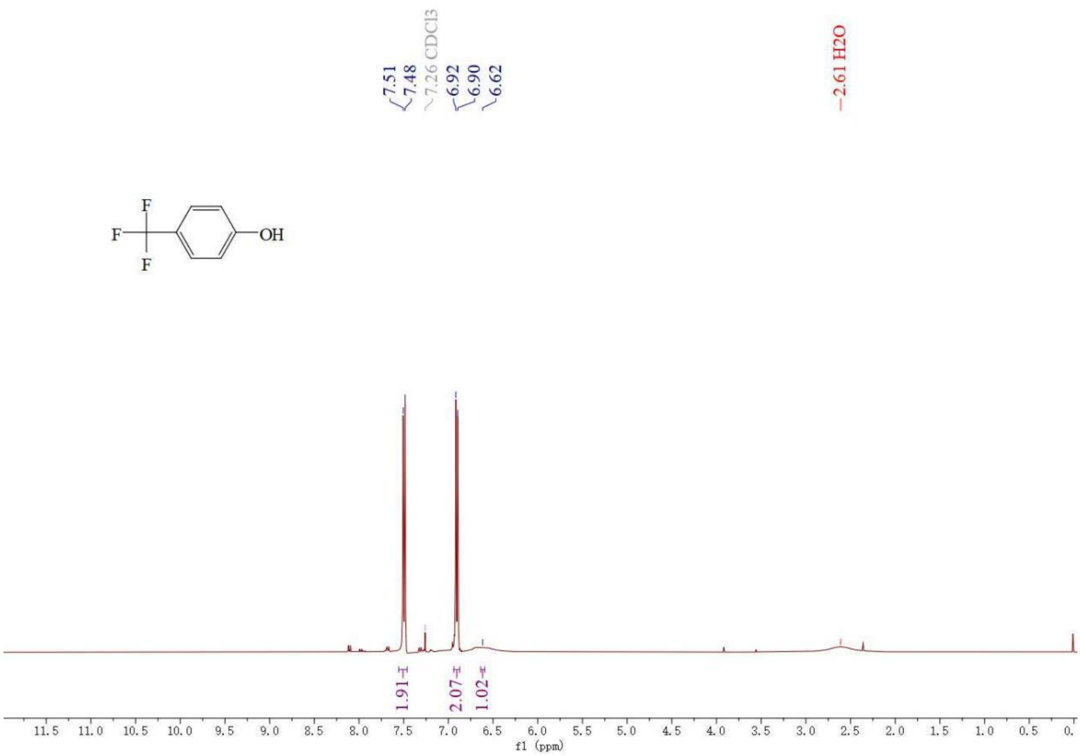

Figure S15. ^{1}HNMR spectrum of isolated product **7b** (400 MHz, chloroform-*d*)

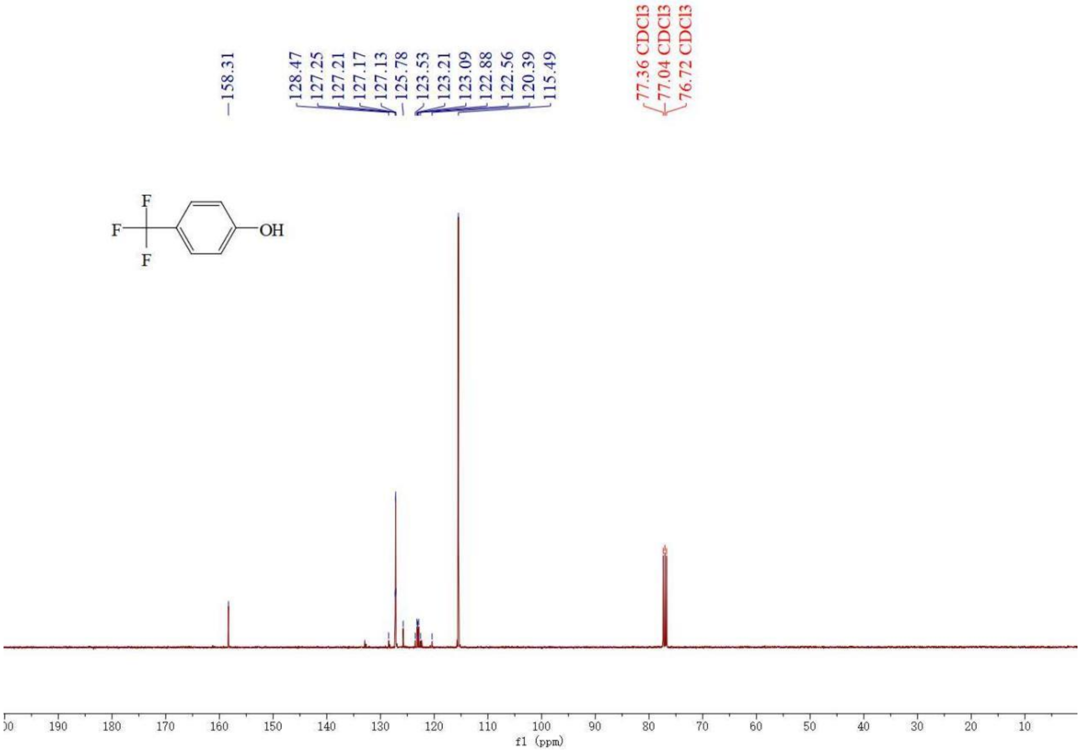

Figure S16. ^{13}CNMR spectrum of isolated product **7b** (100 MHz, chloroform-*d*)

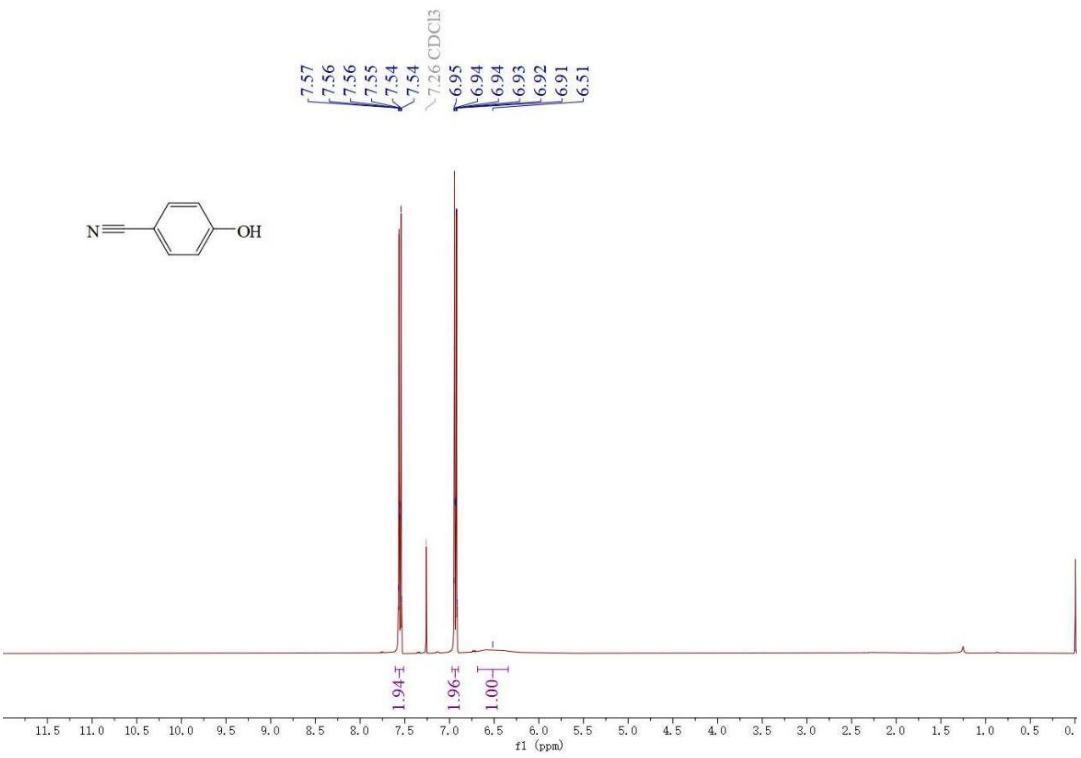

Figure S17. ^{1}HNMR spectrum of isolated product **8b** (400 MHz, chloroform-*d*)

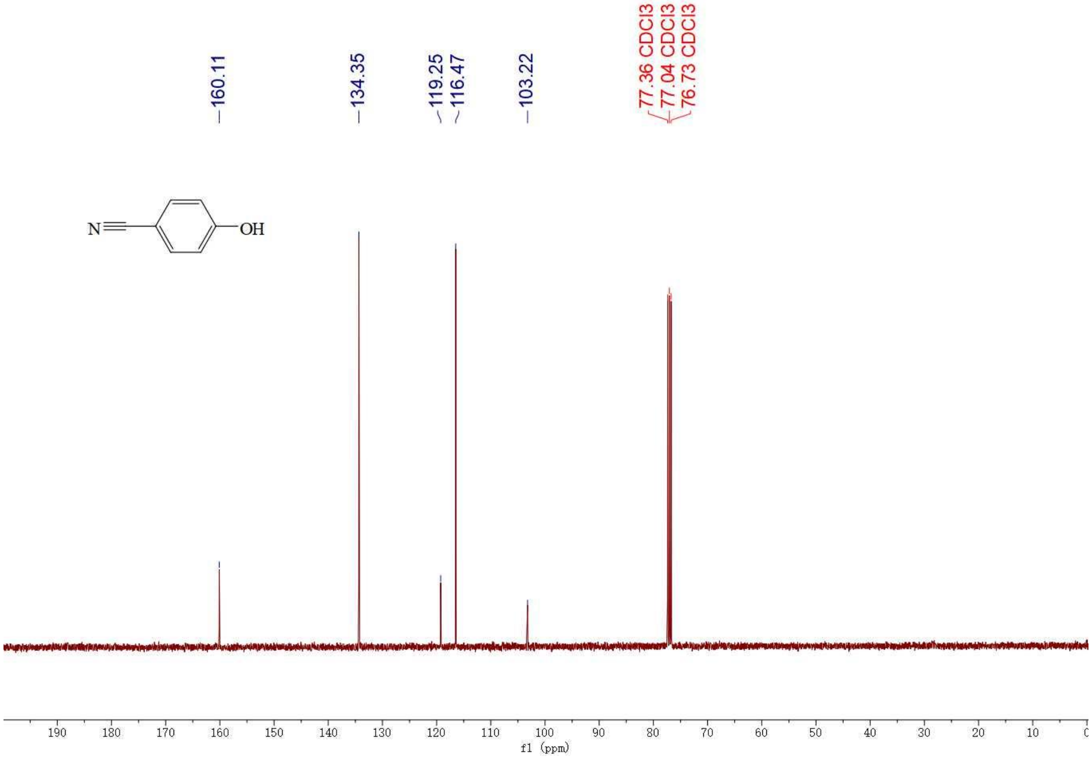

Figure S18. ^{13}CNMR spectrum of isolated product **8b** (100 MHz, chloroform-*d*)

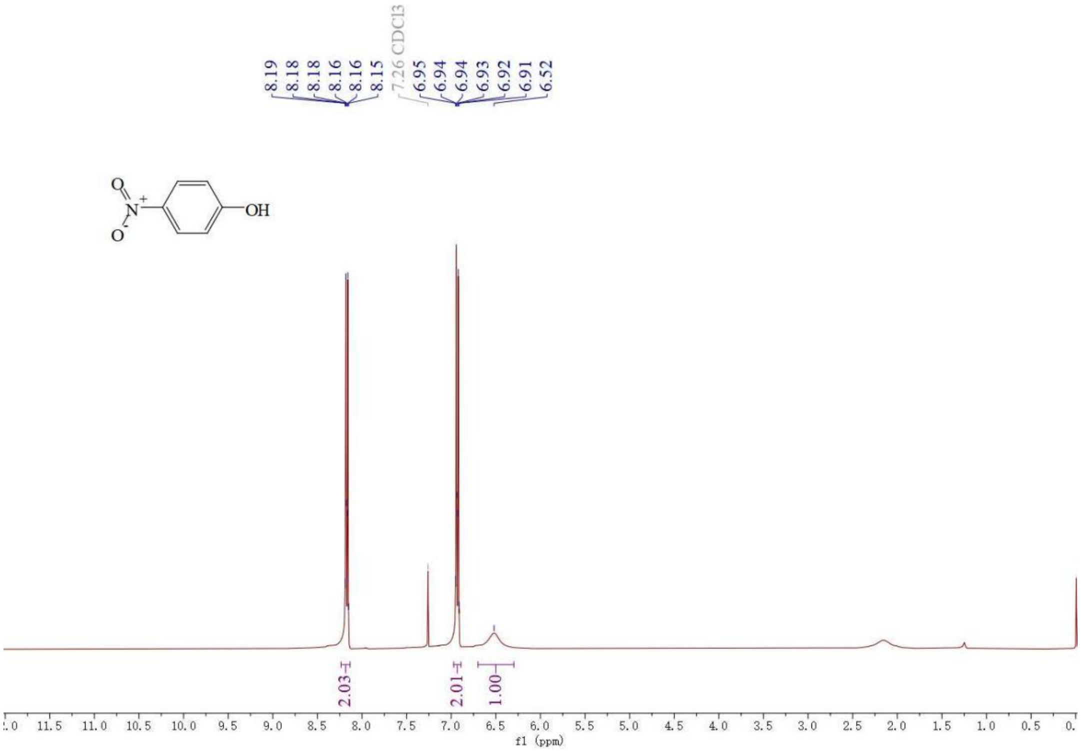

Figure S19. ^{1}HNMR spectrum of isolated product **9b** (400 MHz, chloroform-*d*)

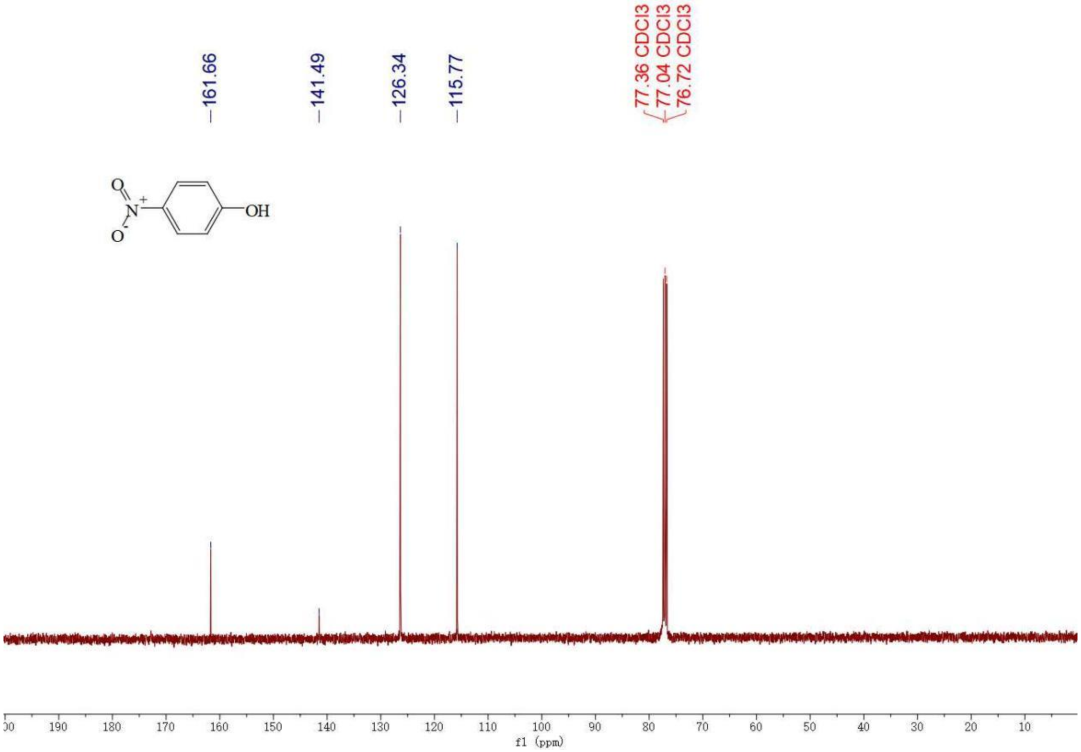

Figure S20. ^{13}CNMR spectrum of isolated product **9b** (100 MHz, chloroform-*d*)

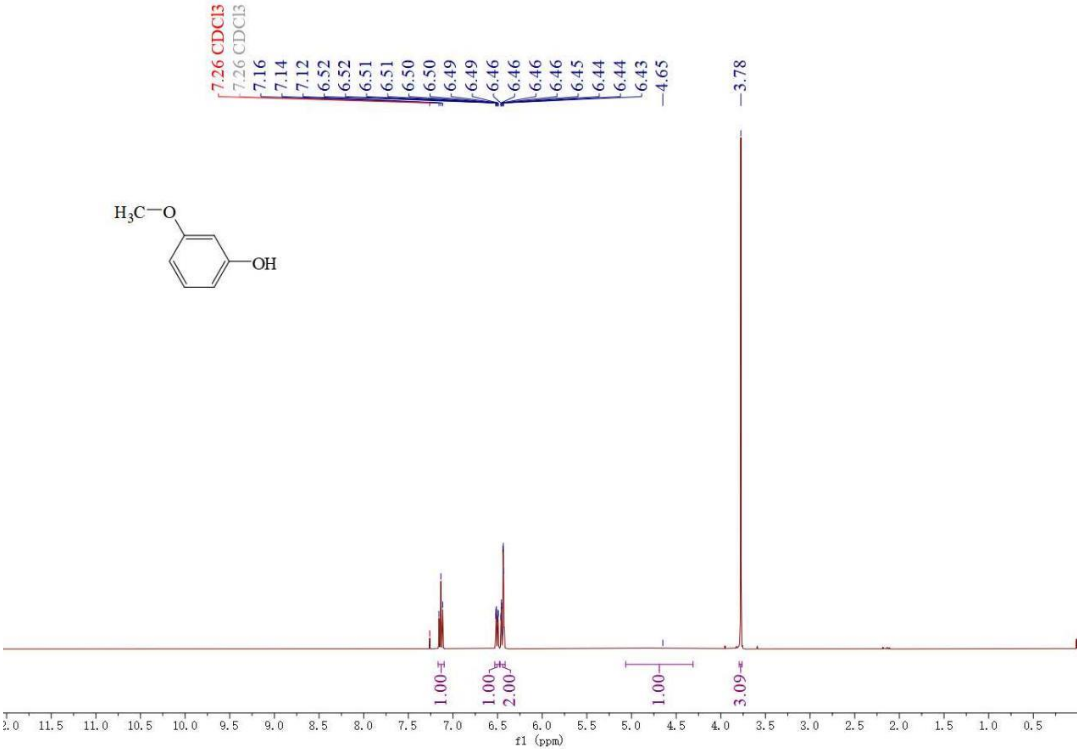

Figure S21. ^{1}HNMR spectrum of isolated product **10b** (400 MHz, chloroform-*d*)

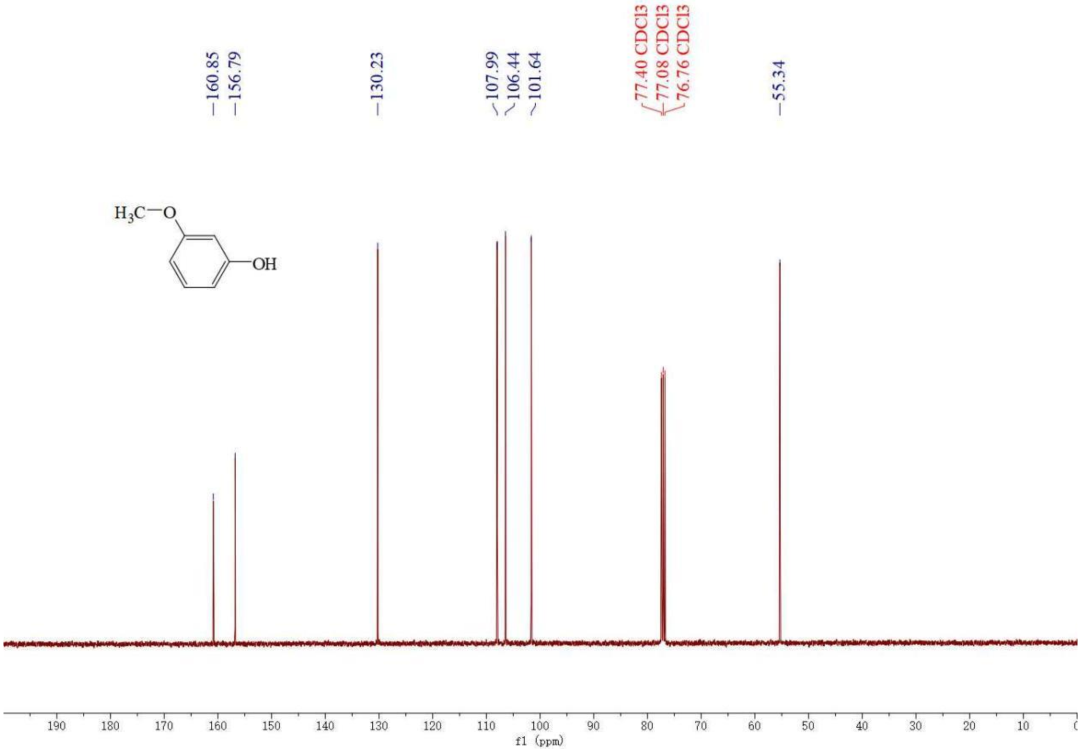

Figure S22. ^{13}CNMR spectrum of isolated product **10b** (100 MHz, chloroform-*d*)

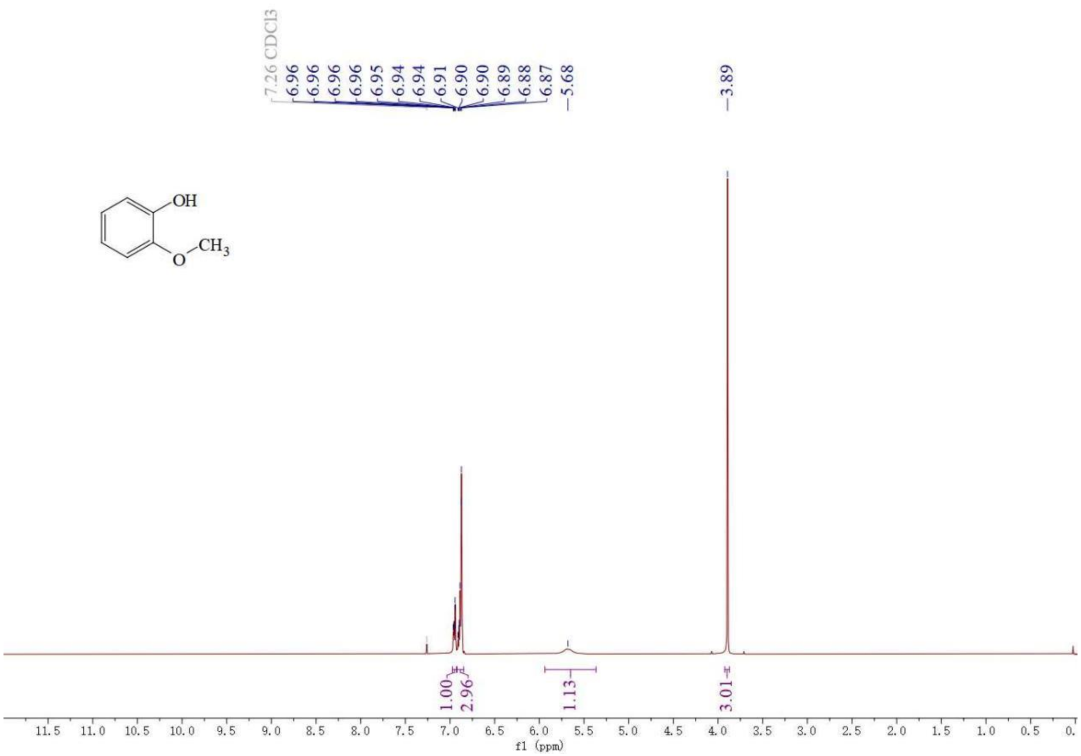

Figure S23. ^{1}HNMR spectrum of isolated product **11b** (400 MHz, chloroform-*d*)

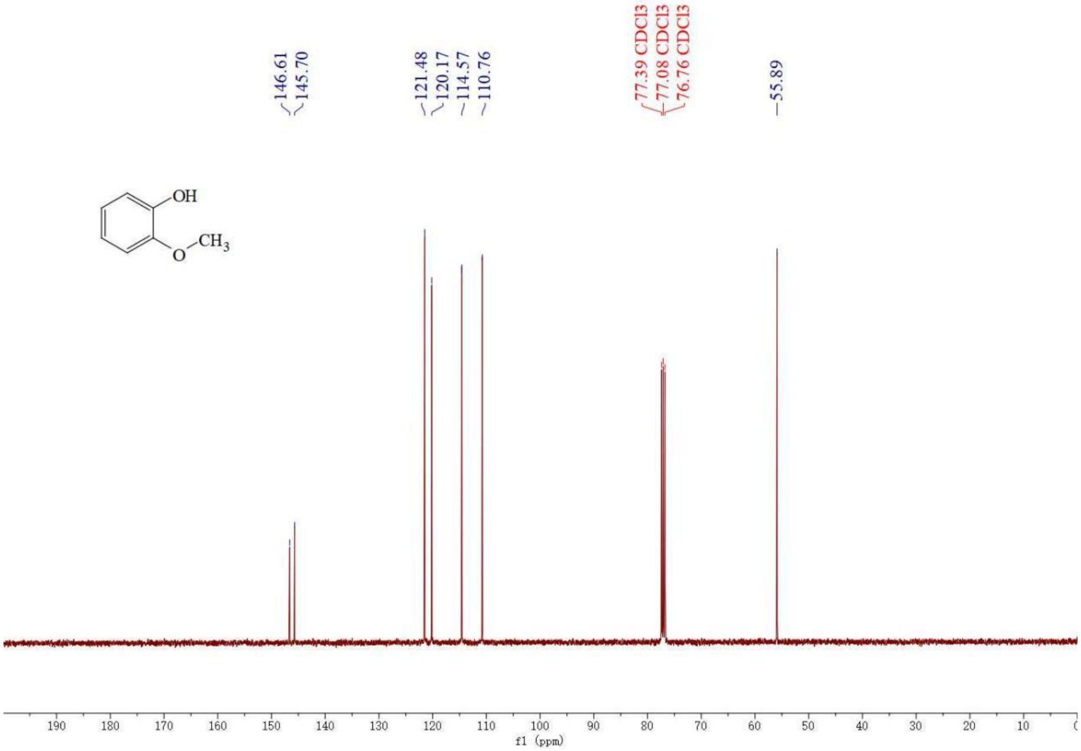

Figure S24. ^{13}CNMR spectrum of isolated product **11b** (100 MHz, chloroform-*d*)

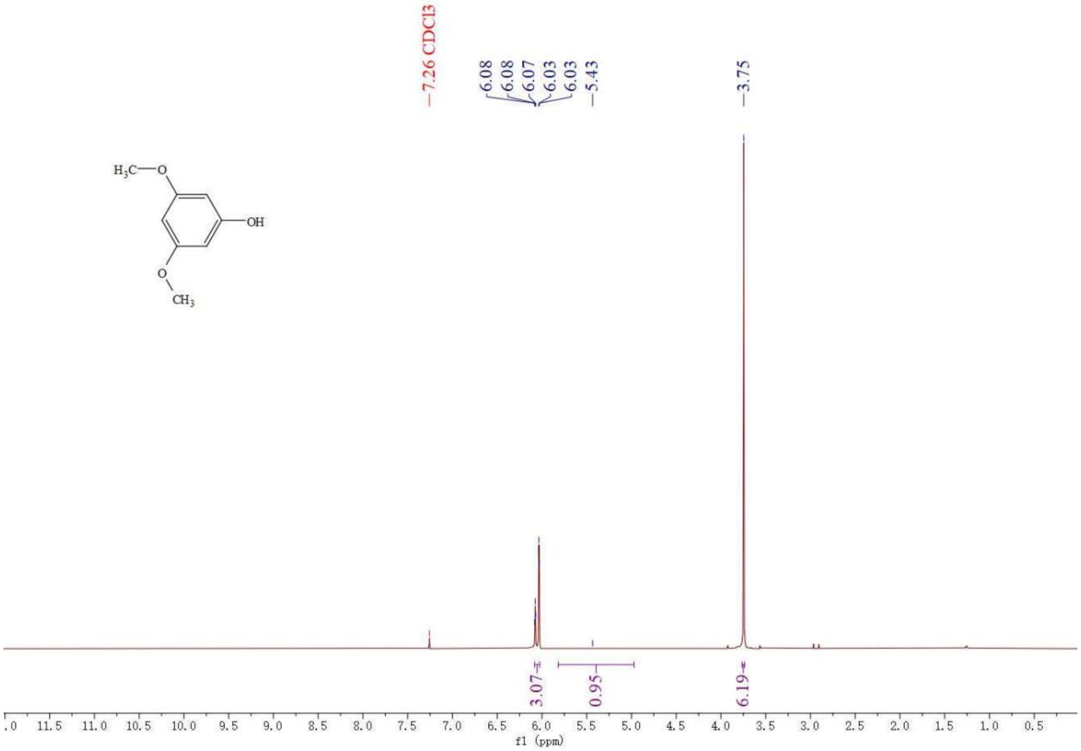

Figure S25. ^{1}HNMR spectrum of isolated product **12b** (400 MHz, chloroform-*d*)

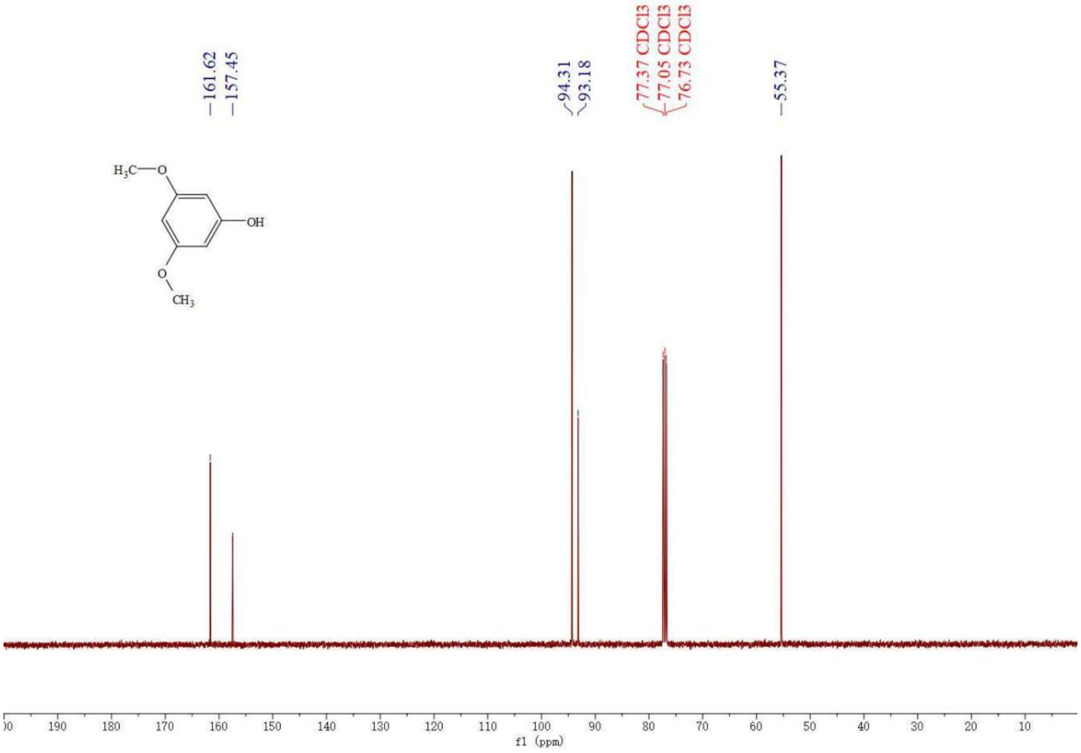

Figure S26. ^{13}CNMR spectrum of isolated product **12b** (100 MHz, chloroform-*d*)

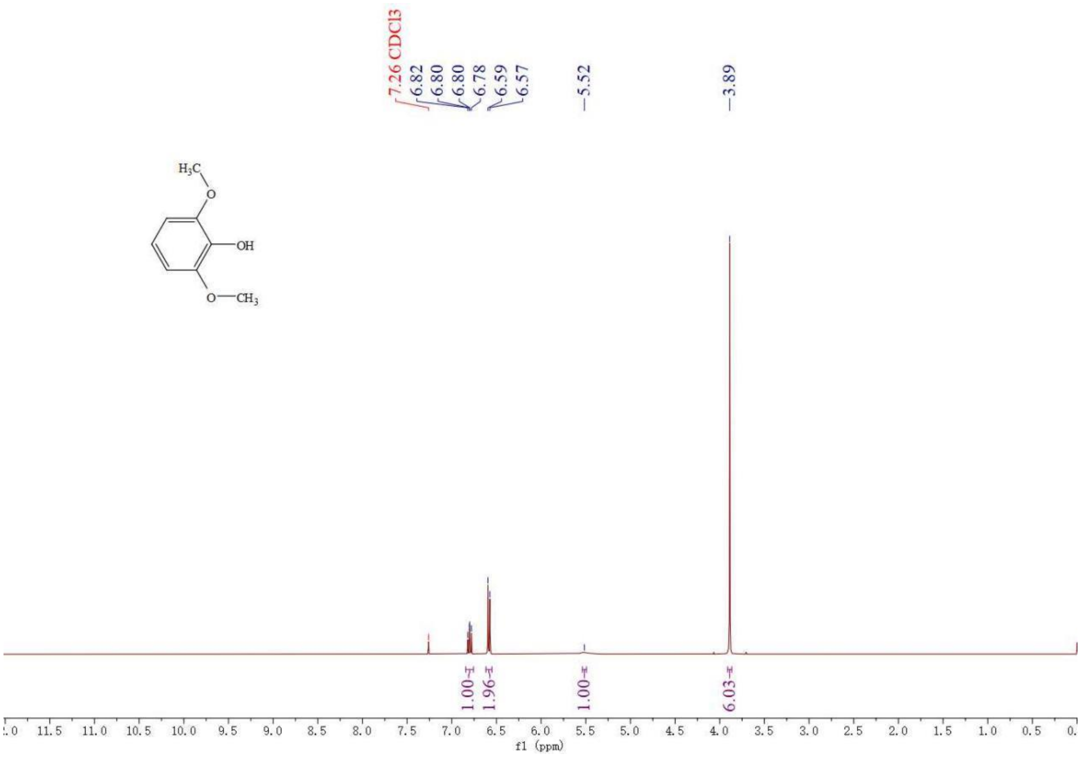

Figure S27. ^{1}HNMR spectrum of isolated product **13b** (400 MHz, chloroform-*d*)

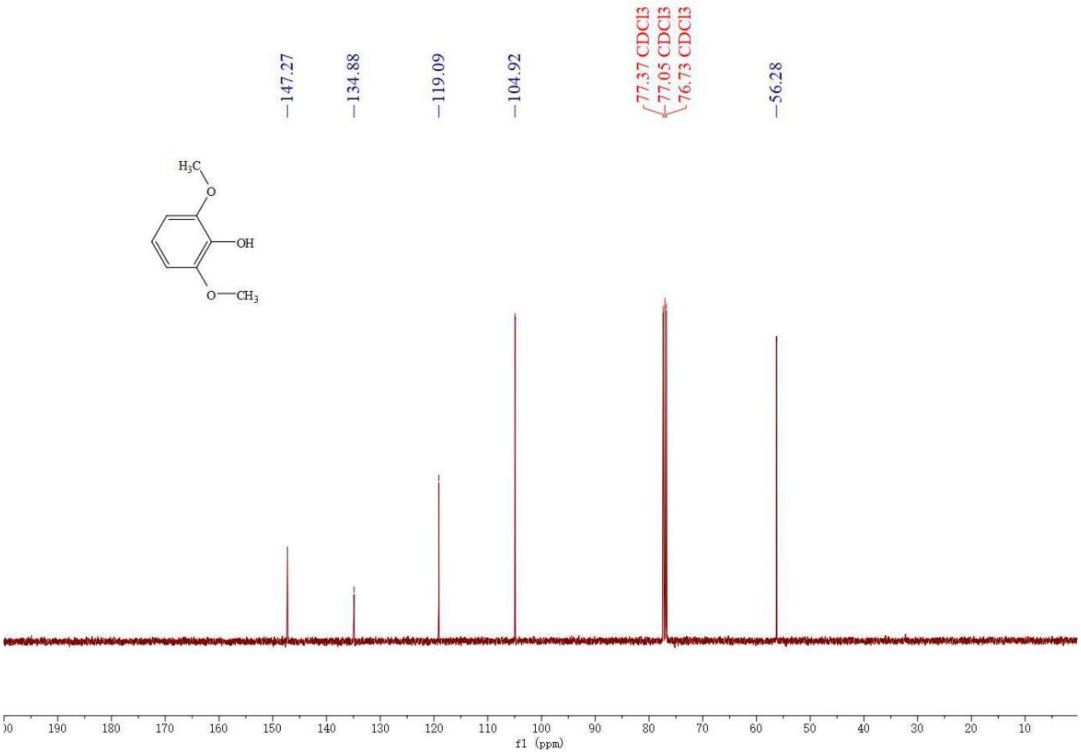

Figure S28. ^{13}CNMR spectrum of isolated product **13b** (100 MHz, chloroform-*d*)

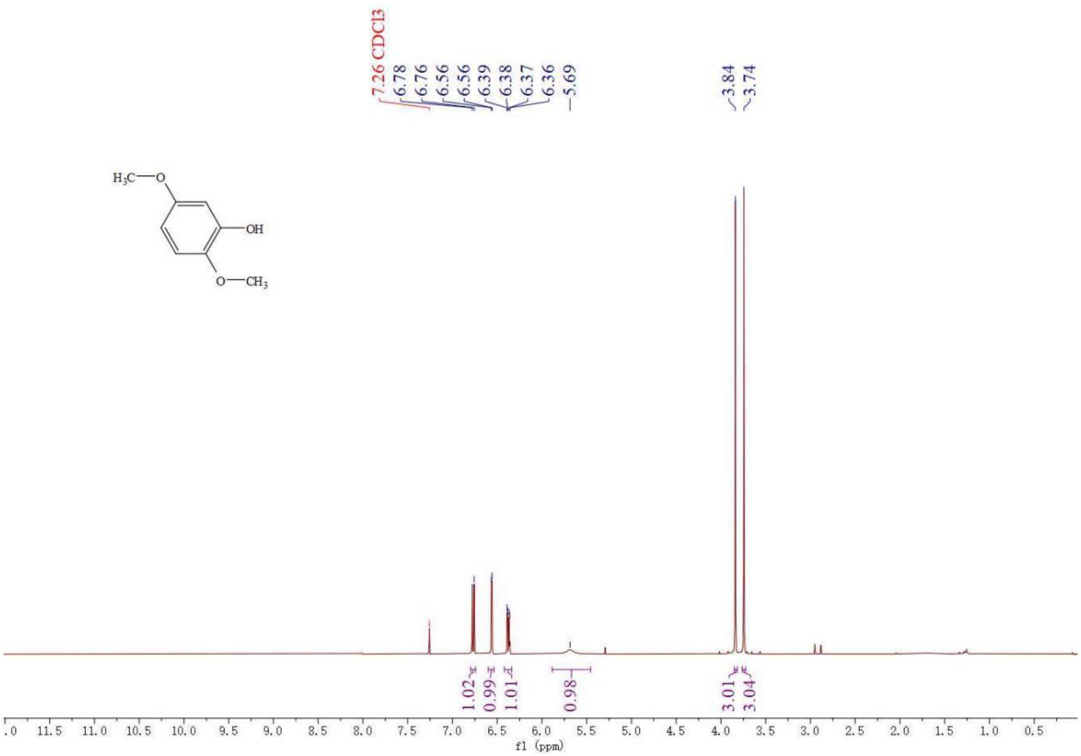

Figure S29. ^{1}HNMR spectrum of isolated product **14b** (400 MHz, chloroform-*d*)

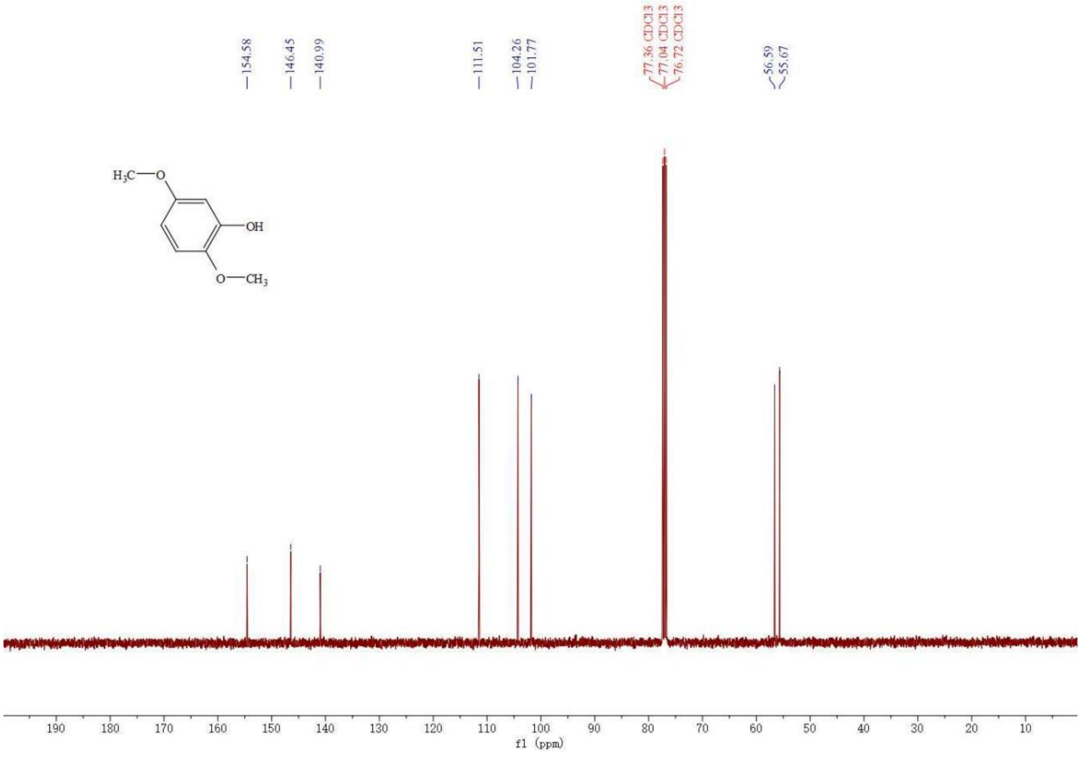

Figure S30. ^{13}CNMR spectrum of isolated product **14b** (100 MHz, chloroform-*d*)

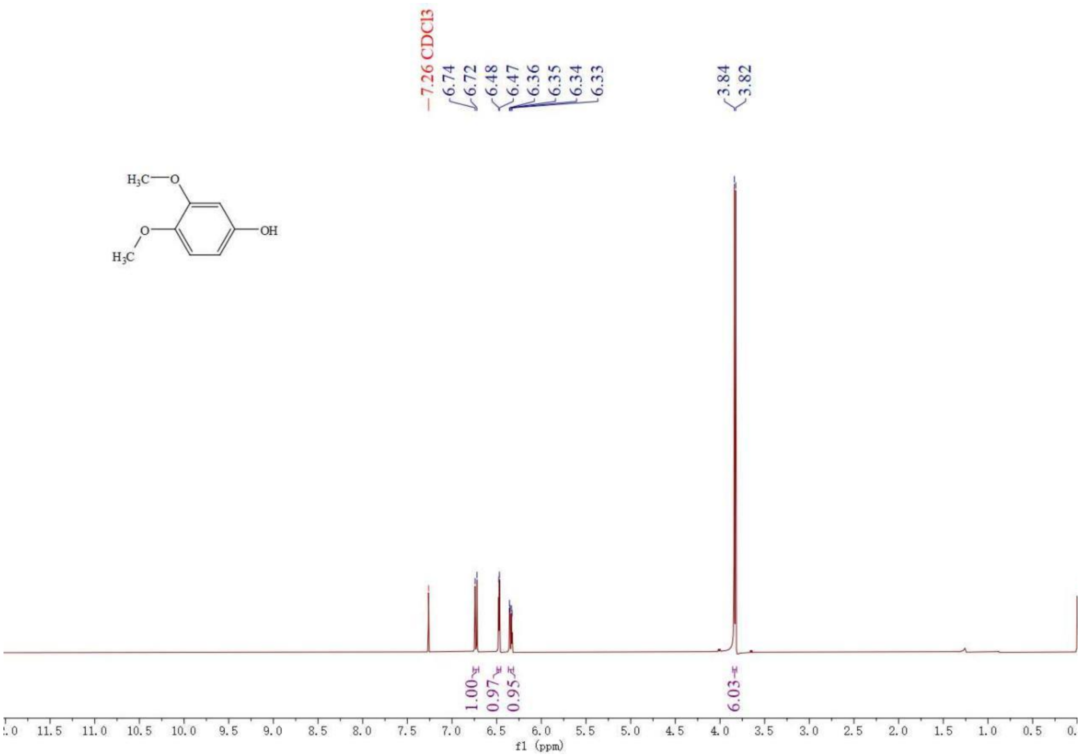

Figure S31. ^{1}HNMR spectrum of isolated product **15b** (400 MHz, chloroform-*d*)

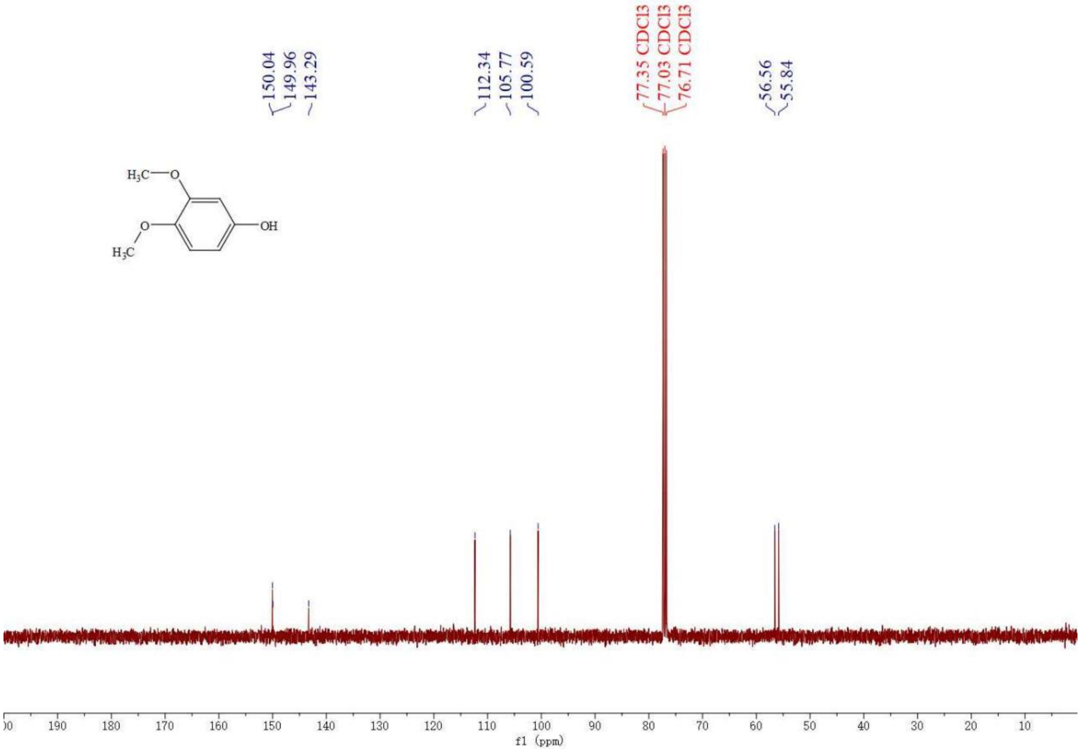

Figure S32. ^{13}CNMR spectrum of isolated product **15b** (100 MHz, chloroform-*d*)

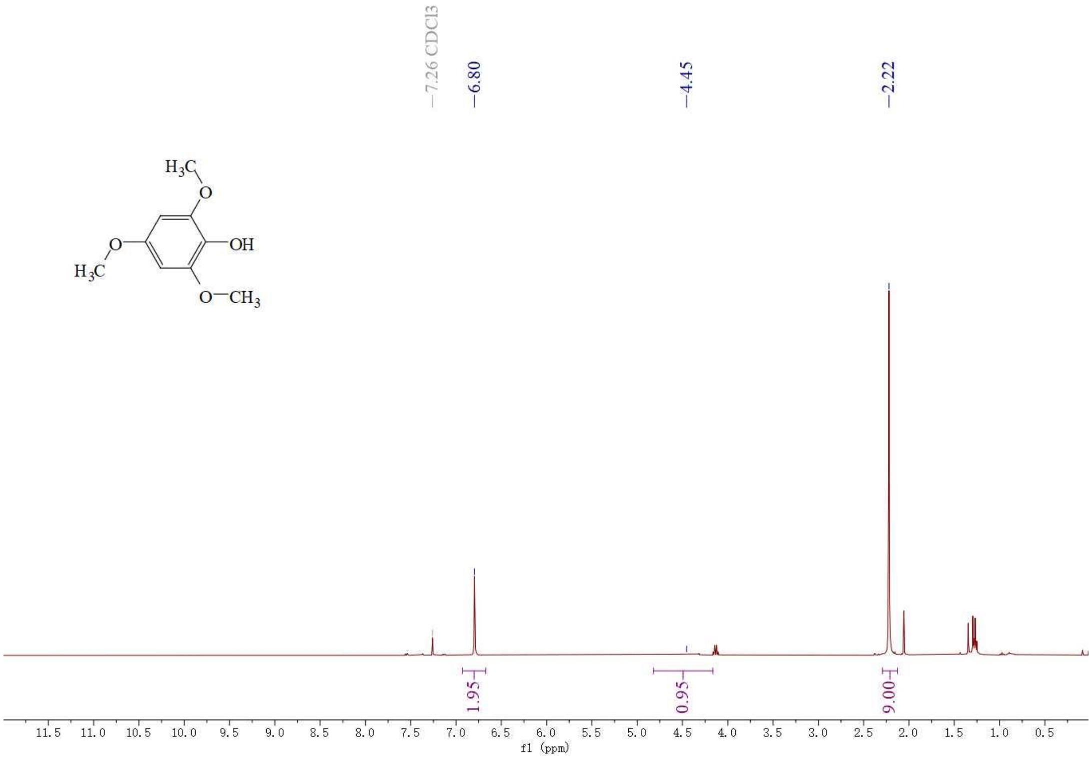

Figure S33. ^{1}HNMR spectrum of isolated product **16b** (400 MHz, chloroform-*d*)

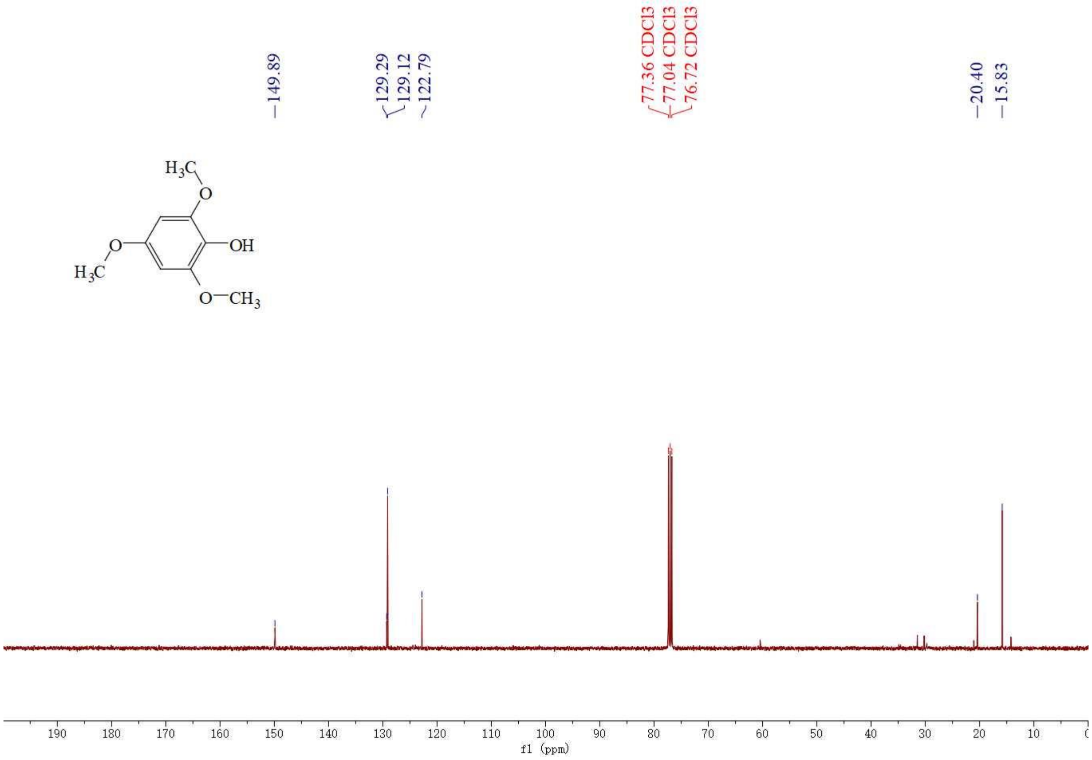

Figure S34. ^{13}CNMR spectrum of isolated product **16b** (100 MHz, chloroform-*d*)

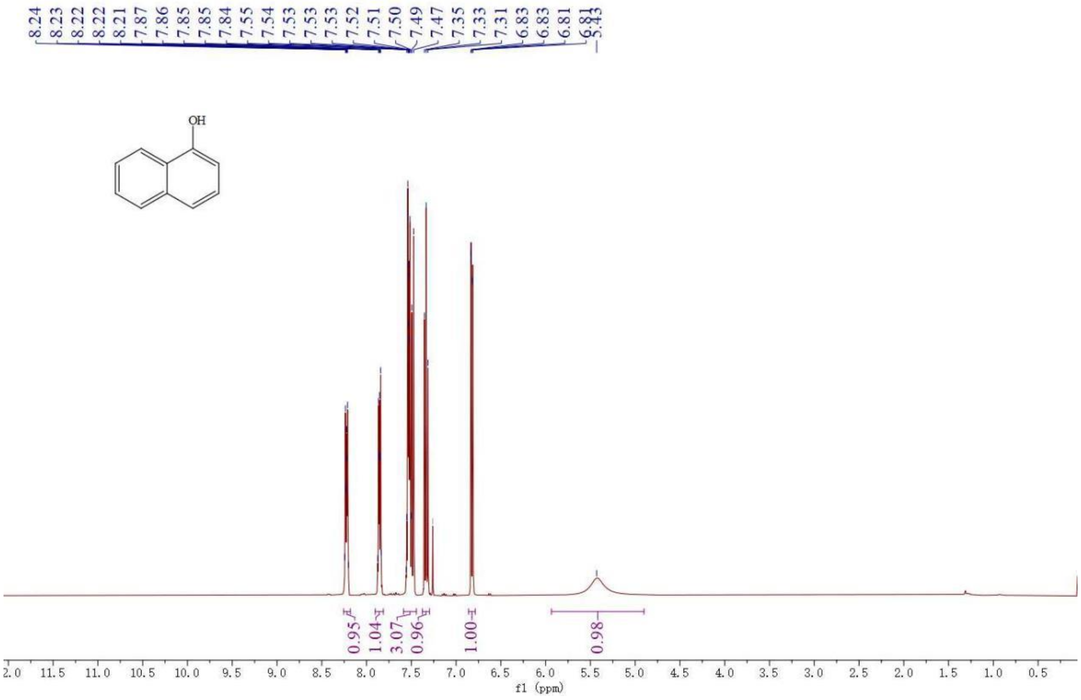

Figure S35. ^{1}HNMR spectrum of isolated product **17b** (400 MHz, chloroform-*d*)

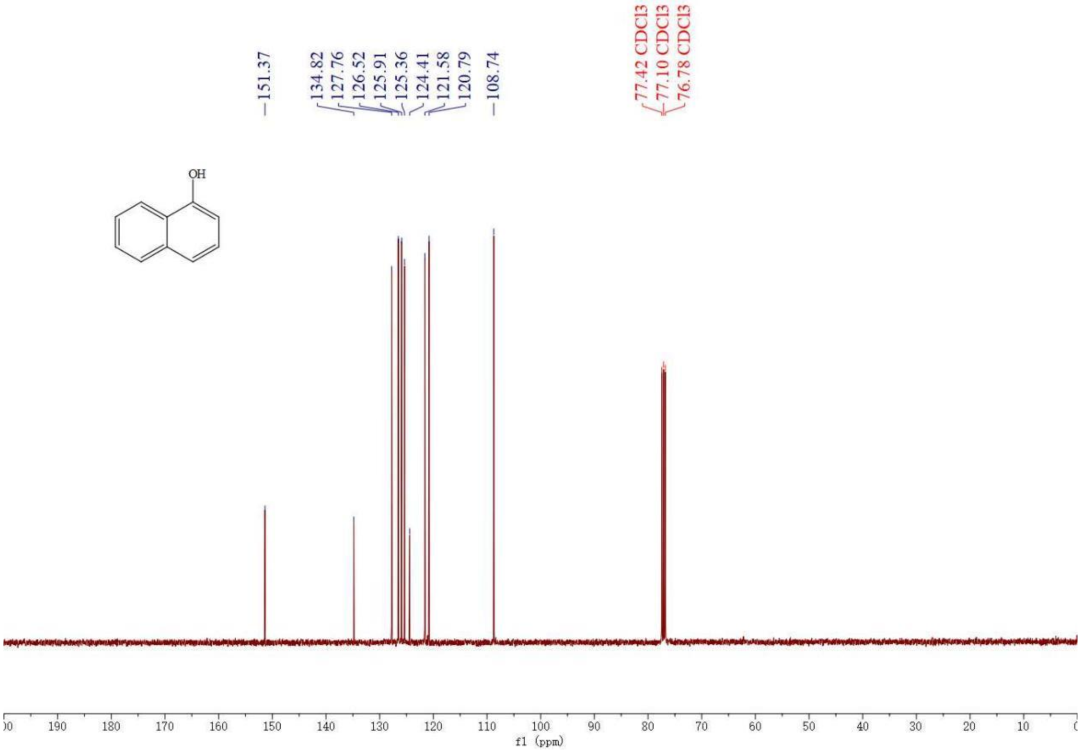

Figure S36. ^{13}CNMR spectrum of isolated product **17b** (100 MHz, chloroform-*d*)

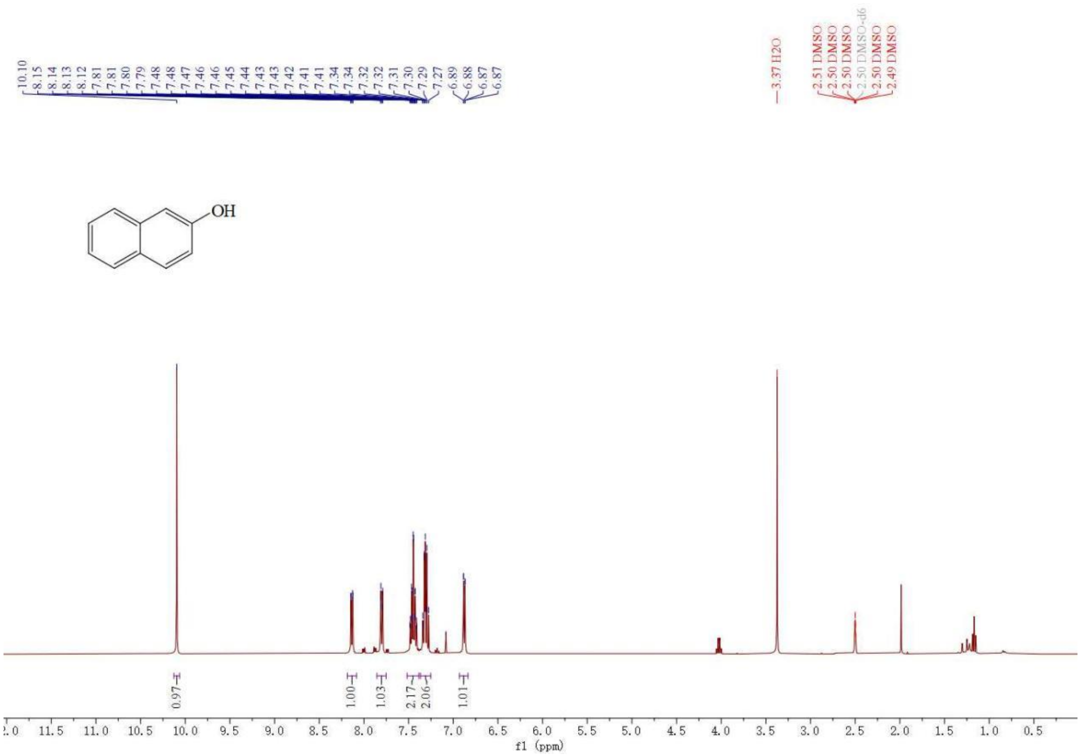

Figure S37. ^{1}HNMR spectrum of isolated product **18b** (400 MHz, DMSO-d_6)

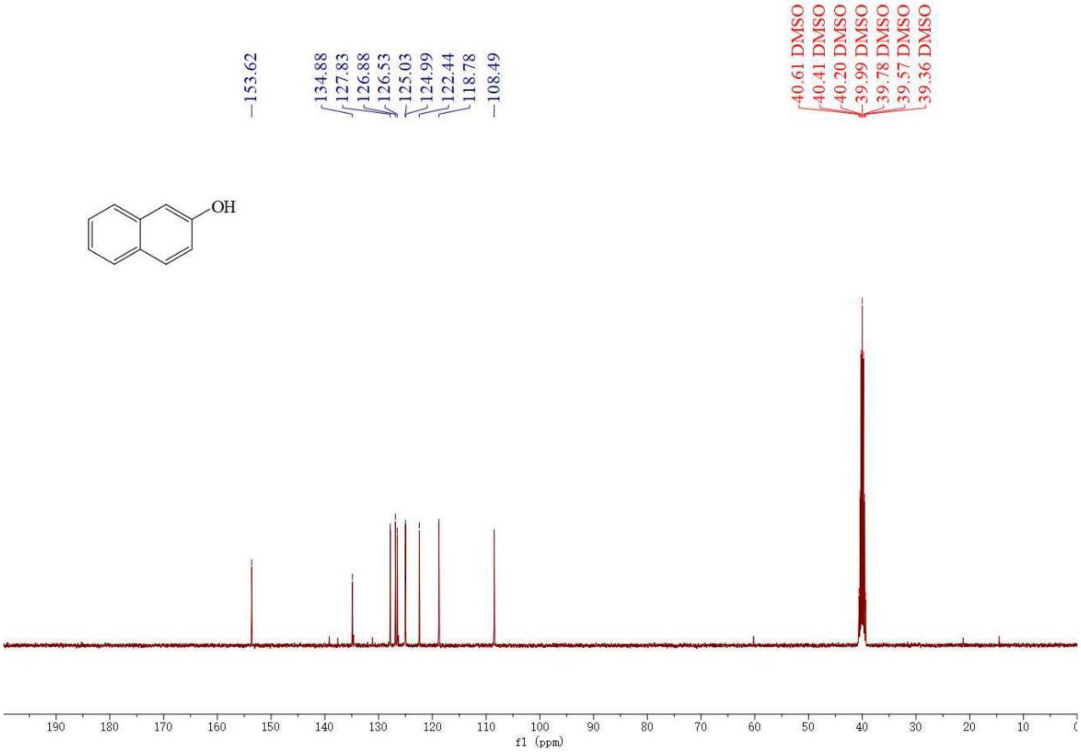

Figure S38. ^{13}CNMR spectrum of isolated product **18b** (100 MHz, DMSO-d_6)

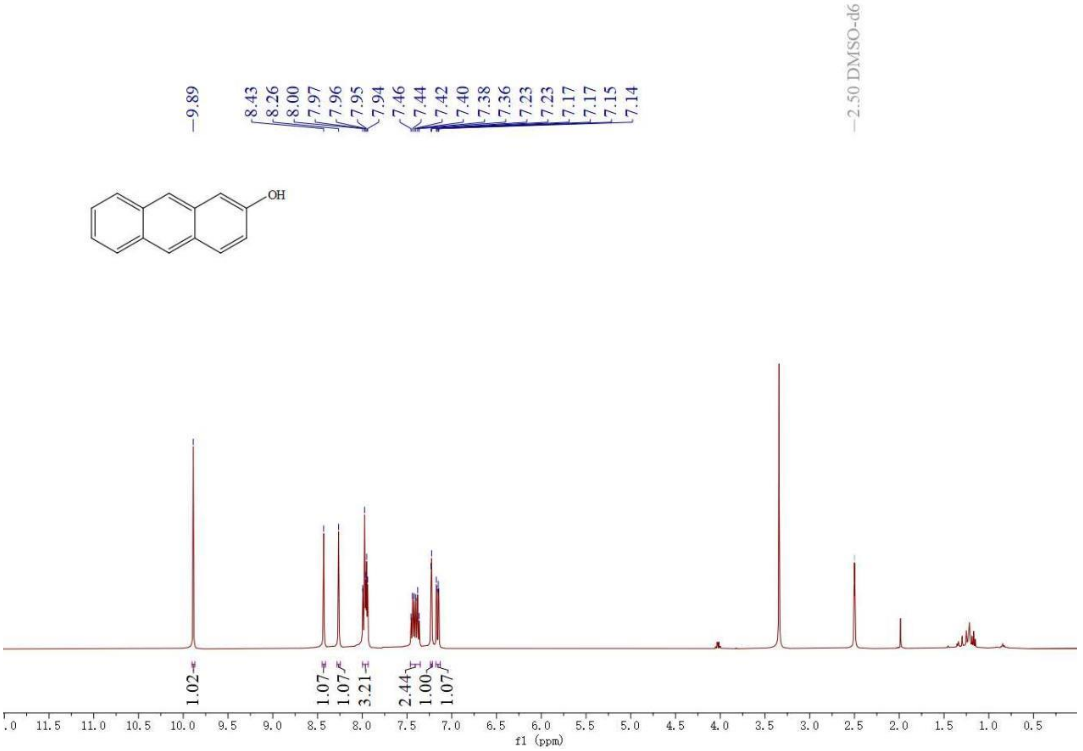

Figure S39. ^{1}HNMR spectrum of isolated product **20b** (400 MHz, DMSO-d_6)

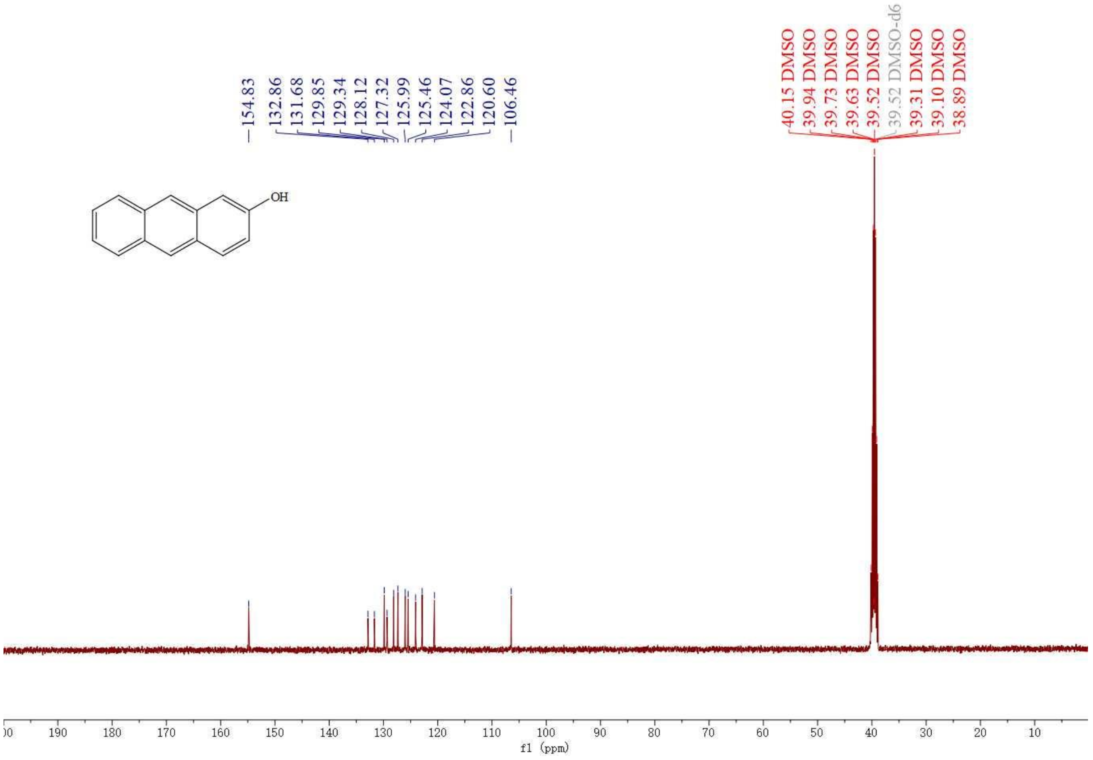

Figure S40. ^{13}CNMR spectrum of isolated product **20b** (100 MHz, DMSO-d_6)

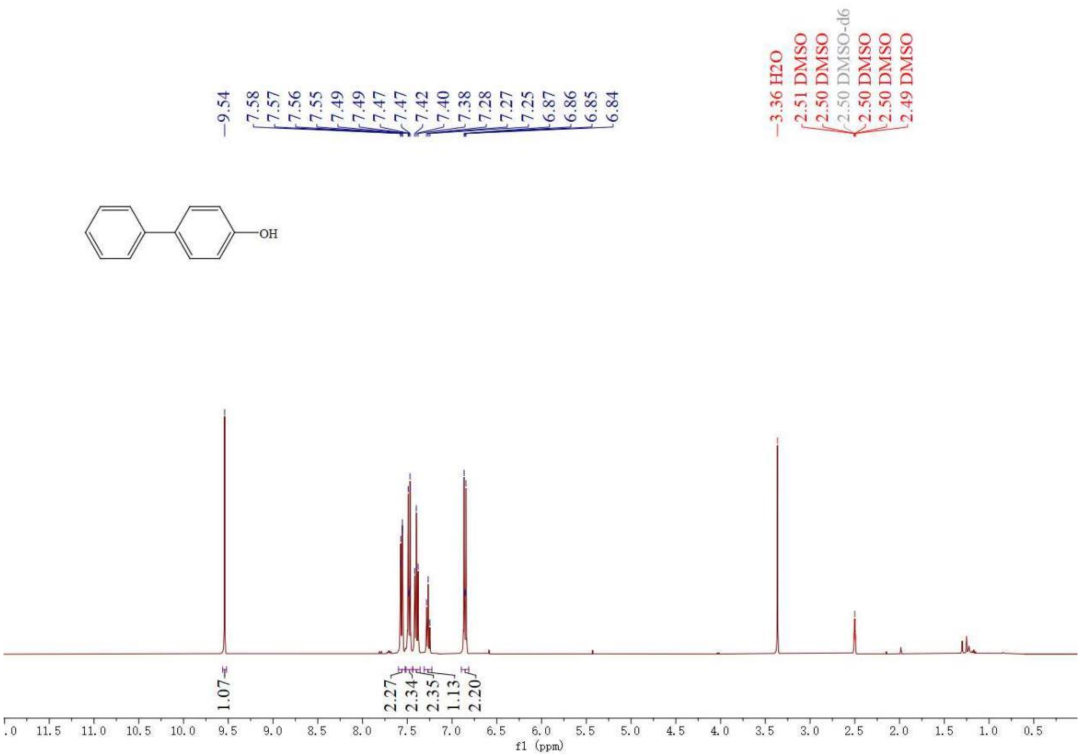

Figure S41. ^{1}HNMR spectrum of isolated product **21b** (400 MHz, DMSO-d$_6$)

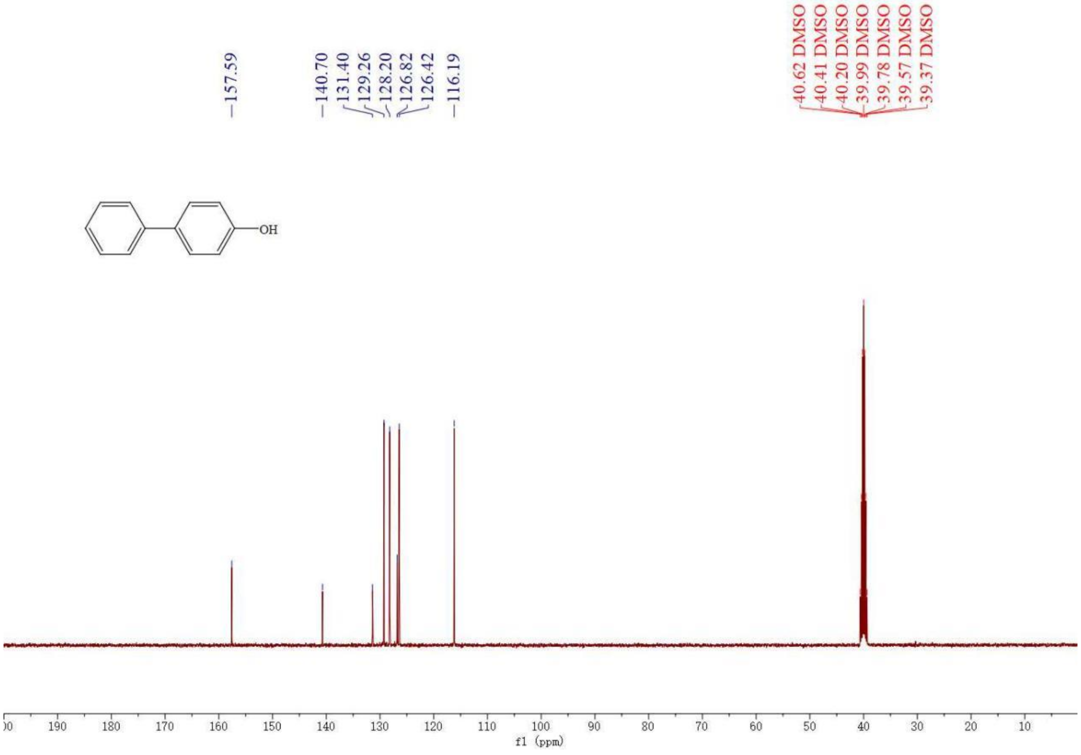

Figure S42. ^{13}CNMR spectrum of isolated product **21b** (100 MHz, DMSO-d$_6$)

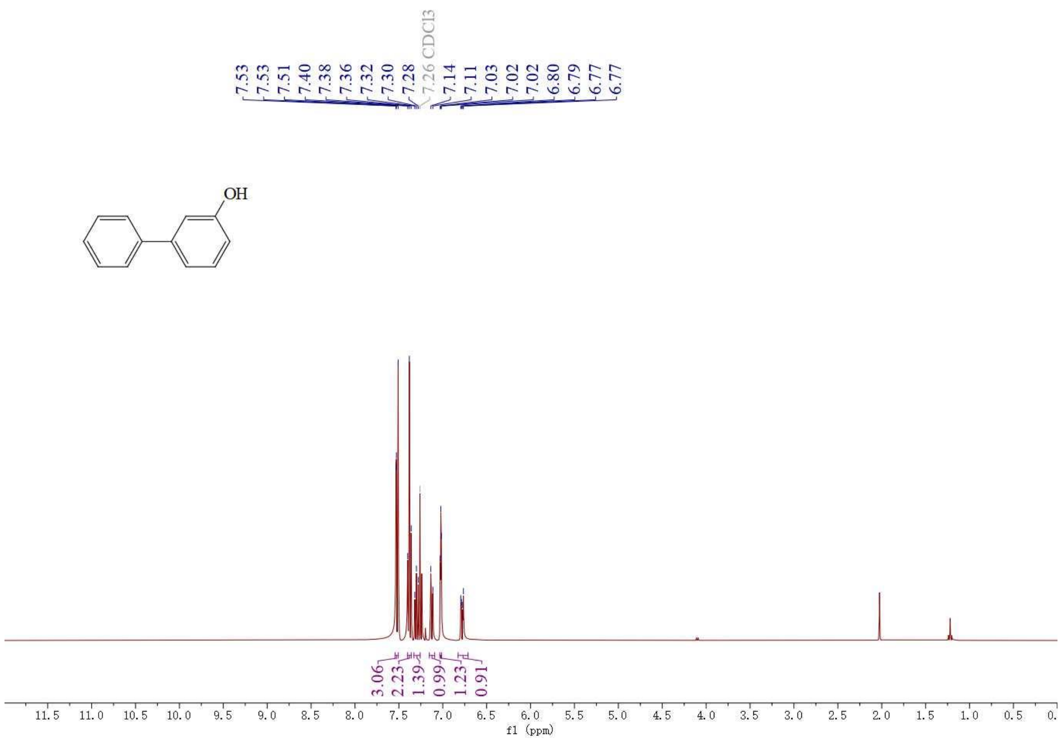

Figure S43. ^{1}HNMR spectrum of isolated product **22b** (400 MHz, chloroform-*d*)

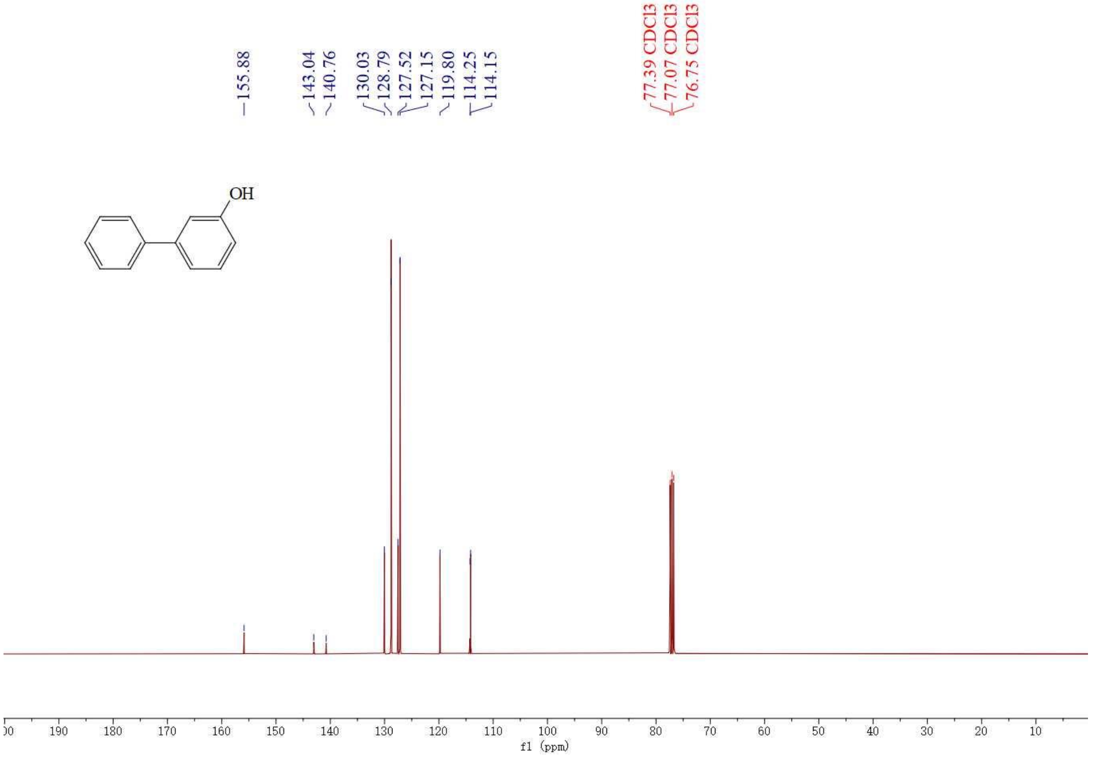

Figure S44. ^{13}CNMR spectrum of isolated product 22**b** (100 MHz, chloroform-*d*)

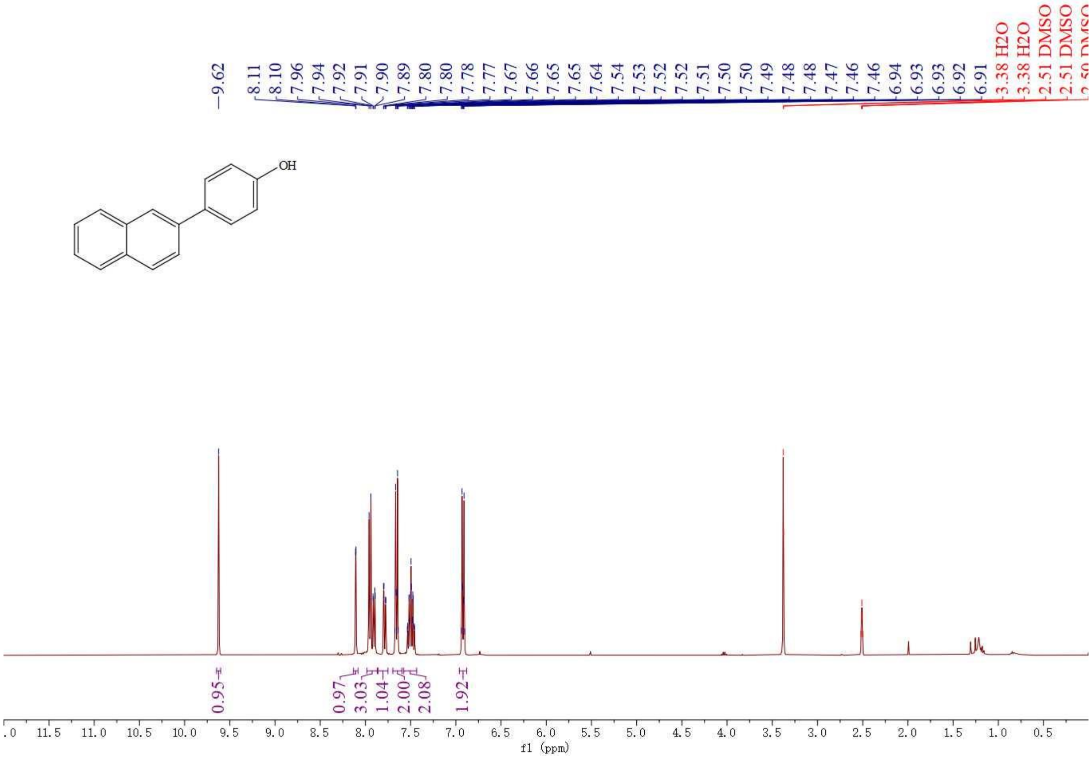

Figure S45. ^{1}HNMR spectrum of isolated product **22b** (400 MHz, DMSO-d_6)

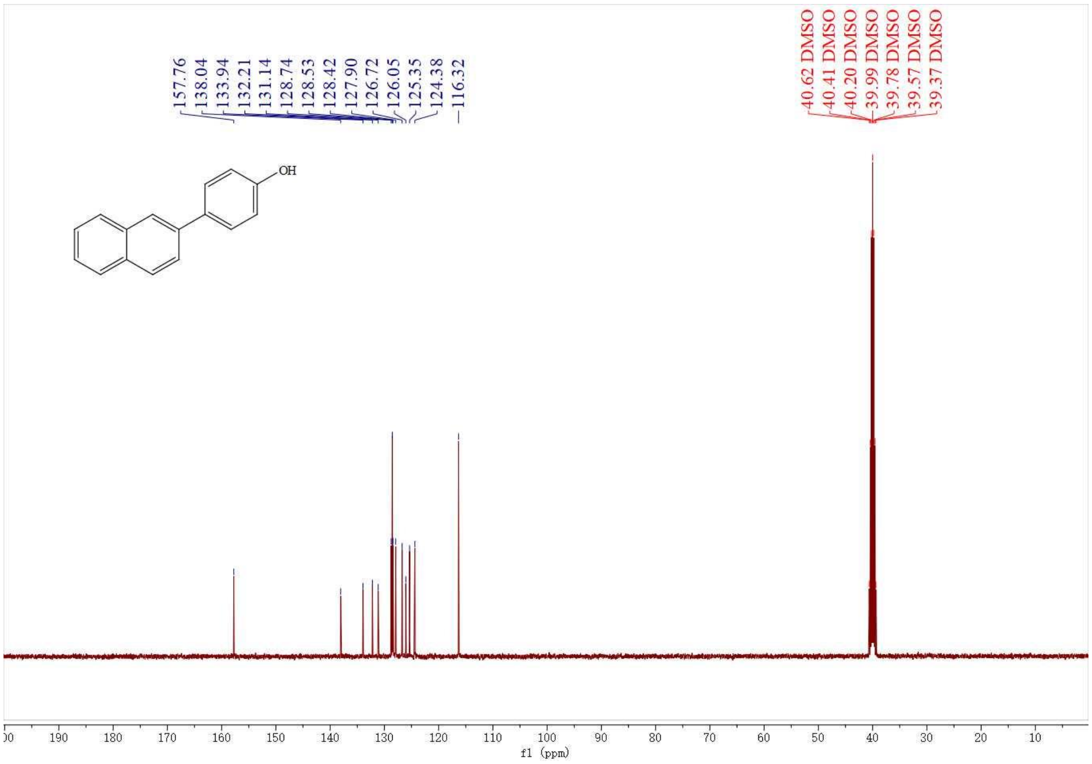

Figure S46. ^{13}CNMR spectrum of isolated product **22b** (100 MHz, DMSO-d_6)

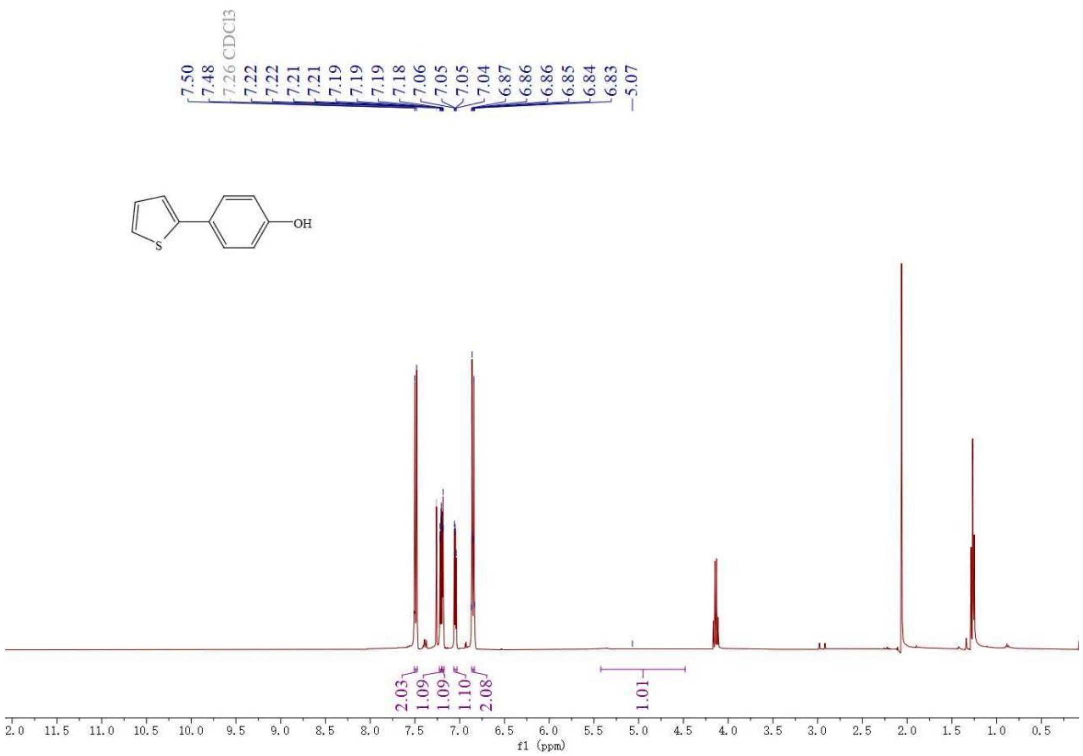

Figure S47. ^{1}HNMR spectrum of isolated product **23b** (400 MHz, DMSO-d_6)

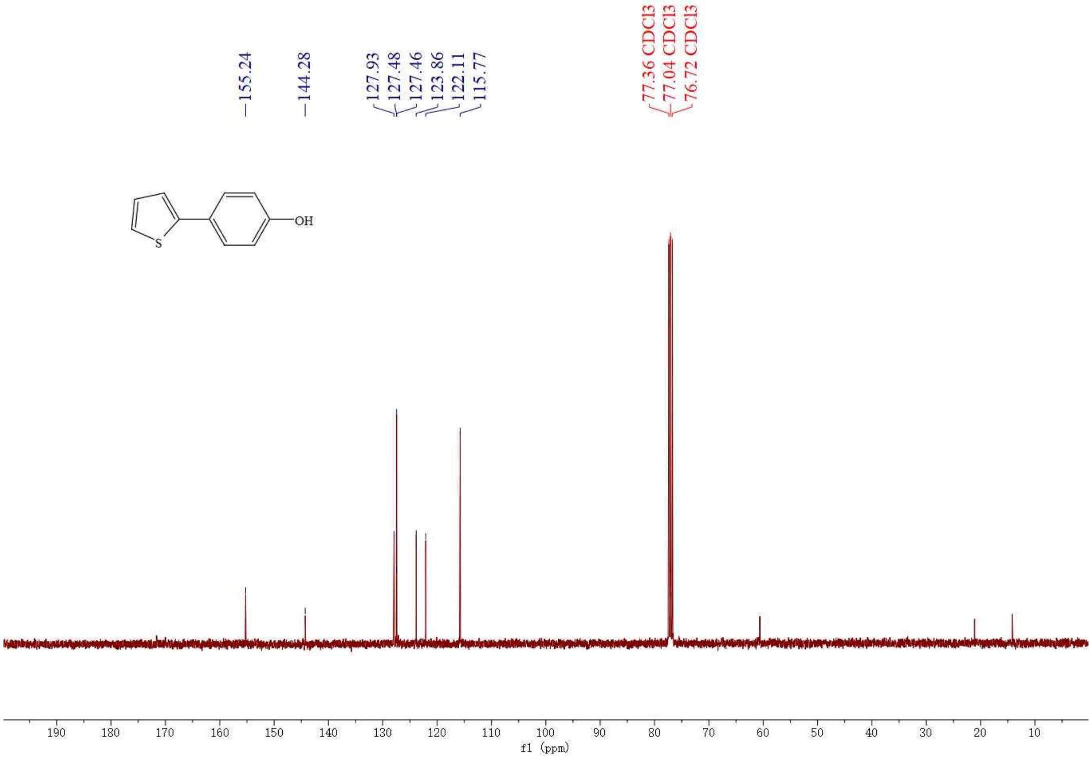

Figure S48. ^{13}CNMR spectrum of isolated product **23b** (100 MHz, DMSO-d_6)

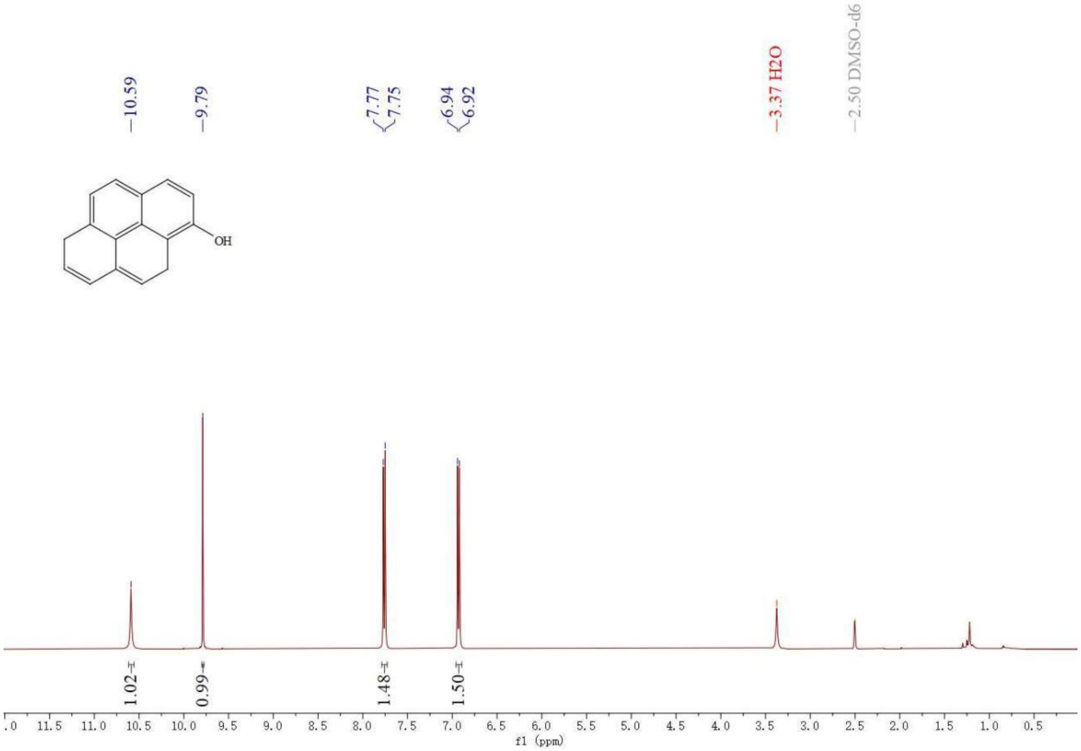

Figure S49. ^{1}HNMR spectrum of isolated product **27b** (400 MHz, DMSO-d_6)

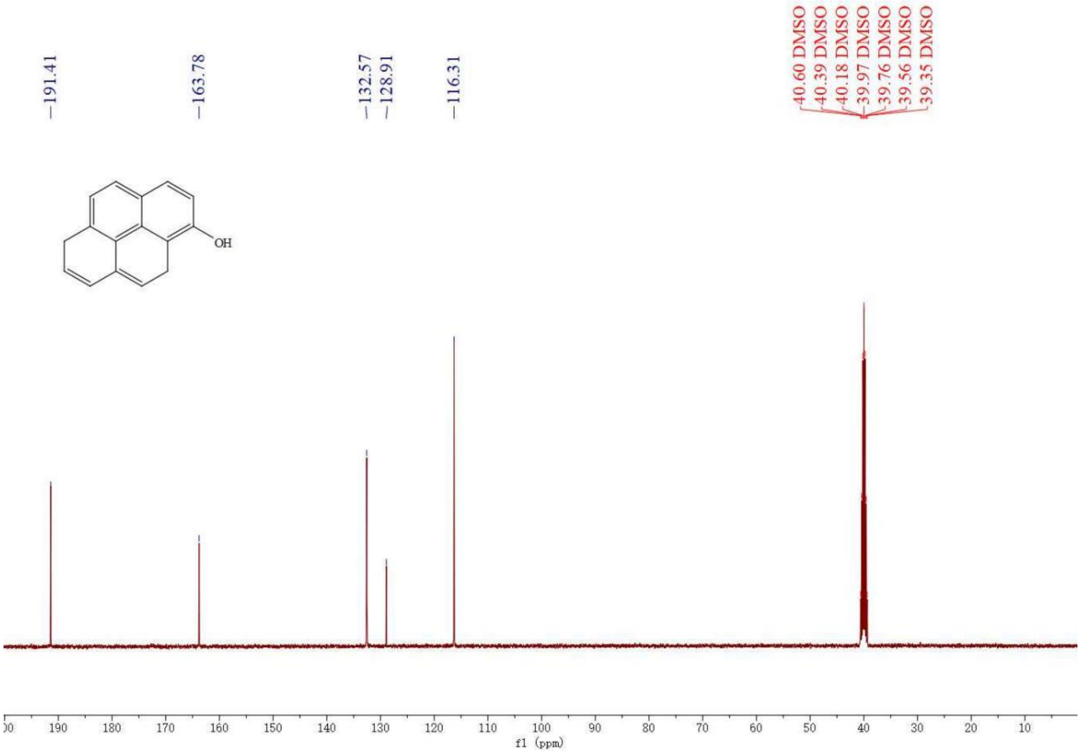

Figure S50. ^{13}CNMR spectrum of isolated product **27b** (100 MHz, DMSO-d_6)

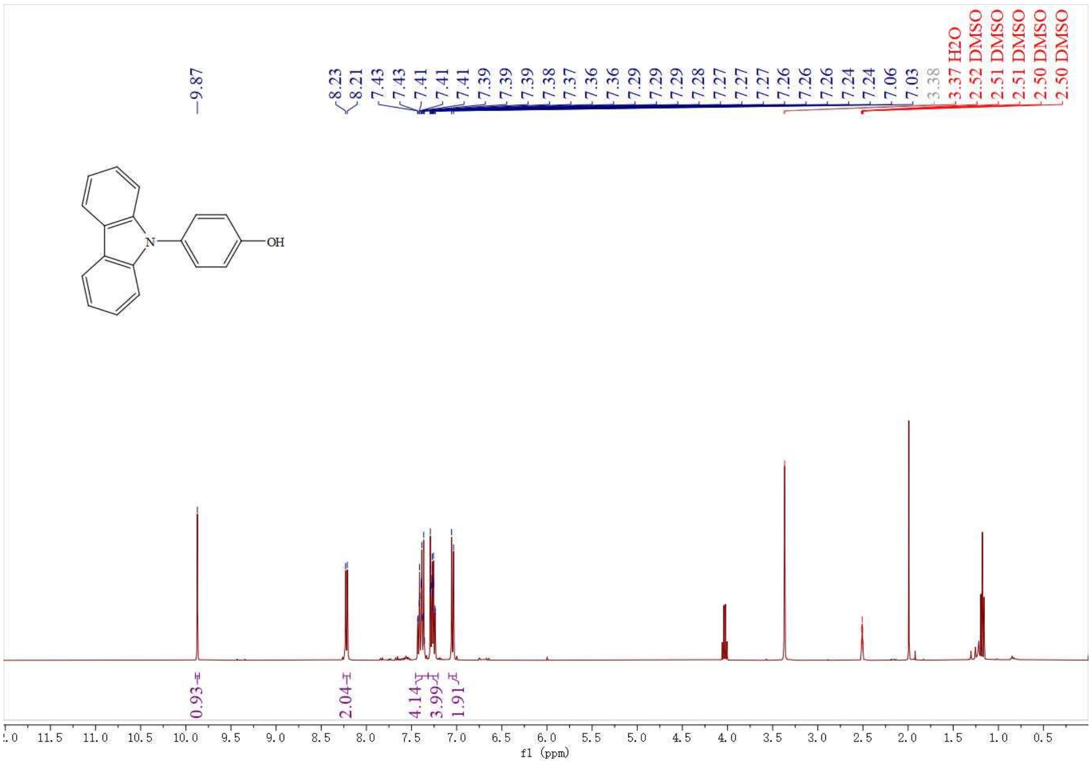

Figure S51. ^{1}HNMR spectrum of isolated product **28b** (400 MHz, DMSO-d_6)

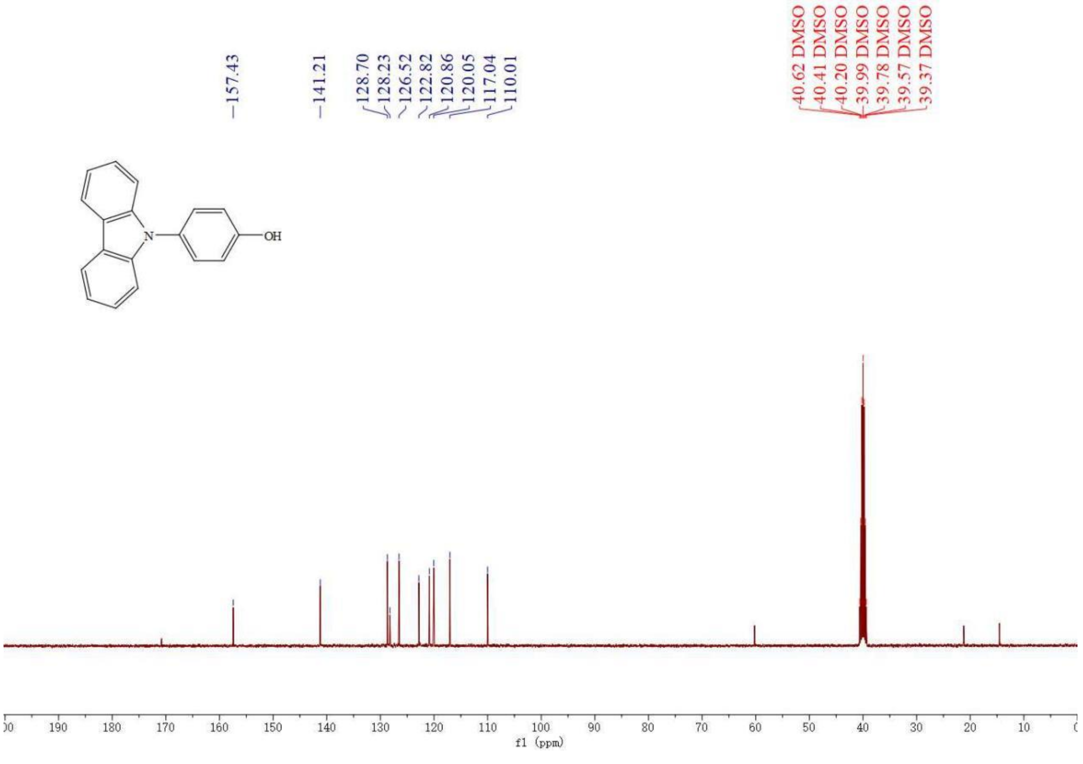

Figure S52. ^{13}CNMR spectrum of isolated product **28b** (100 MHz, DMSO-d_6)

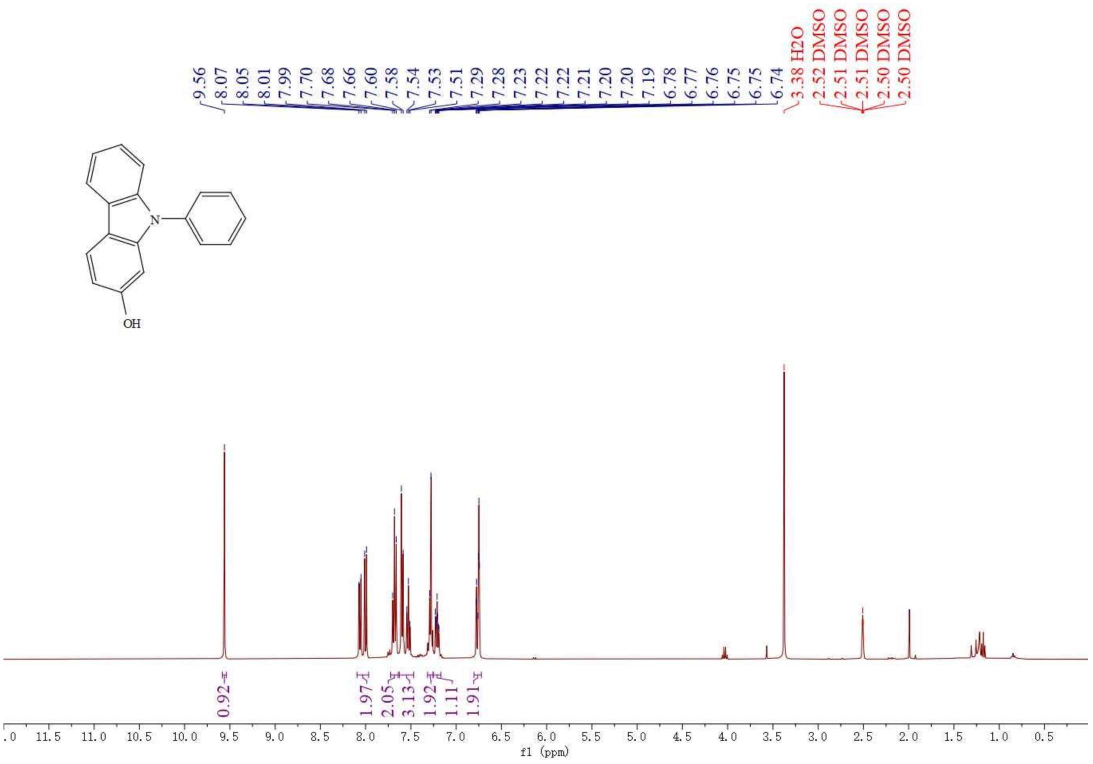

Figure S53. ^{1}HNMR spectrum of isolated product **29b** (400 MHz, DMSO-d_6)

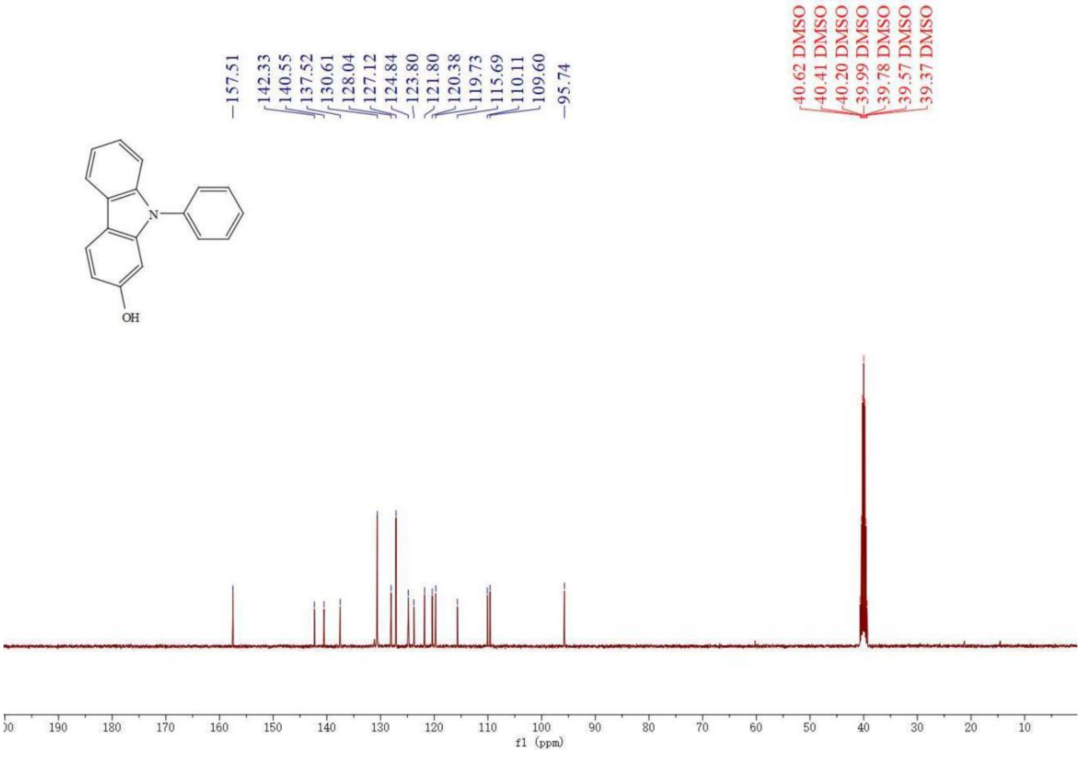

Figure S54. ^{13}CNMR spectrum of isolated product **29b** (100 MHz, DMSO-d_6)
